# New insights on *Prestosuchus chiniquensis* Huene, 1942 (Pseudosuchia, Loricata) based on new specimens from the “Tree Sanga” Outcrop, Chiniquá Region, Rio Grande do Sul, Brazil

**DOI:** 10.7717/peerj.1622

**Published:** 2016-02-01

**Authors:** Marcel B. Lacerda, Bianca M. Mastrantonio, Daniel C. Fortier, Cesar L. Schultz

**Affiliations:** 1 Instituto de Geociências, Laboratório de Paleovertebrados, Universidade Federal do Rio Grande do Sul–UFRGS, Porto Alegre, Rio Grande do Sul, Brazil; 2 CHNUFPI, Campus Amílcar Ferreira Sobral, Universidade Federal do Piauí, Floriano, Piauí, Brazil

**Keywords:** Pseudosuchia, Middle triassic, Prestosuchus, Santa Maria Supersequence, Ontogeny

## Abstract

The ‘rauisuchians’ are a group of Triassic pseudosuchian archosaurs that displayed a near global distribution. Their problematic taxonomic resolution comes from the fact that most taxa are represented only by a few and/or mostly incomplete specimens. In the last few decades, renewed interest in early archosaur evolution has helped to clarify some of these problems, but further studies on the taxonomic and paleobiological aspects are still needed. In the present work, we describe new material attributed to the ‘rauisuchian’ taxon *Prestosuchus chiniquensis*, of the *Dinodontosaurus* Assemblage Zone, Middle Triassic (Ladinian) of the Santa Maria Supersequence of southern Brazil, based on a comparative osteologic analysis. Additionally, we present well supported evidence that these represent juvenile forms, due to differences in osteological features (*i.e.*, a subnarial fenestra) that when compared to previously described specimens can be attributed to ontogeny and indicate variation within a single taxon of a problematic but important osteological structure in the study of ‘rauisuchians.’

## Introduction

The evolution of archosaurs along the pseudosuchian lineage during the Triassic led to the appearance of many diverse groups that occupied a variety of stages along global trophic webs (Gauthier, 1984; Gauthier & Padian, 1985; Benton & Clark, 1988; Sereno, 1991; Juul, 1994; Brusatte et al., 2010; Nesbitt, 2011). Although some taxa can be assigned to clearly monophyletic groups, such as aetosaurs (*e.g.* Nesbitt, 2011; Desojo et al., 2013), a number of forms that historically did not fit within the less inclusive clades were assigned to the ‘Rauisuchia.’ This group is traditionally composed of taxa that possess a variety of body plans, from large-bodied quadruped predators (*e.g. Prestosuchus chiniquensis* Huene, 1942, *Saurosuchus galilei Reig,* 1959, *Fasolasuchus tenax* Bonaparte, 1981) to small cursorial, bipedal, endentulous forms (*e.g. Shuvosaurus inexpectatus* (Chatterjee, 1993), *Effigia okeeffeae* (Nesbitt & Norell, 2006)) but due to shared cranial traits (additional openings in the dermatocranium) and similarities in hindlimb, pelvic and ankle morphologies (Gower, 2000; Nesbitt et al., 2013) were “bunched” together. There is no current consensus on the taxonomic definition of “Rauisuchia” and the content of the different proposed subgroups (Rauisuchidae, Prestosuchidae, Poposauridae, Chatterjeeidae) has varied (Gower, 2000; Nesbitt, 2011; Nesbitt et al., 2013). Most phylogenetic studies lack a consensus on the stability of the group, since there are no clear ‘rauisuchian’ apomorphies (Gower, 2000; Nesbitt et al., 2013). Among the proposed hypotheses, some have considered them as monophyletic (Brusatte et al., 2010; França, Ferigolo & Langer, 2011) or paraphyletic (Benton & Clark, 1988; Parrish, 1993; Juul, 1994; Weinbaum & Hungerbühler, 2007; Gauthier et al., 2011; Nesbitt, 2011; Butler et al., 2011), with recent works discussing the abandonment of the name altogether (see detailed discussion in Nesbitt et al. (2013)). The problematic alpha taxonomy of this group is due, in part, to the incomplete condition of many of the described specimens (Gower, 2000; Nesbitt, 2011; Nesbitt et al., 2013). However problematic, “Rauisuchia” is still used in recent works (*e.g.* Nesbitt et al., 2013), but placed between commas, to reflect the unclear affinities. Since the topic of the present article is beyond this problem, we will follow this trend in referring to this group to simplify the presentation of our work.

‘Rauisuchians’ displayed a temporal range from the Early to Late Triassic (Butler et al., 2011), with their fossils being found in all continents except Antarctica (Benton, 1984; Benton, 1986; Bonaparte, 1984; Gower, 2000; Nesbitt et al., 2013). In South America, they are found in Brazil and Argentina. In the latter, the oldest taxon is *Luperosuchus fractus* (Romer, 1971) from the Chañares Formation (Middle-Late Triassic) (Romer, 1971; Desojo & Arcucci, 2009). Among Late Triassic taxa, *Sillosuchus longicervix* (Alcober & Parrish, 1997) and *Saurosuchus galilei* occur in the Ischigualasto Formation (Sill, 1974; Alcober & Parrish, 1997; Alcober, 2000; Nesbitt, 2011; Trotteyn, Desojo & Alcober, 2011; Nesbitt et al., 2013), whereas *Fasolasuchus tenax* (Bonaparte, 1981) occurs in the Los Colorados Formation (Late Triassic).

In Brazil, all ‘rauisuchian’ taxa are found only in the Pinheiros-Chiniquá and Santa Cruz sequences of Santa Maria Supersequence of the Rio Grande do Sul State (Zerfass et al., 2003; Horn et al., 2014). In this region, during the 1920’s, the German paleontologist Friedrich von Huene and his collaborators discovered the first fossil remains of what he would later designate as ‘rauisuchians.’ The species described were *Rauisuchus tiradentes*, *Prestosuchus loricatus*, *Prestosuchus chiniquensis* and *Procerosuchus celer* (Huene, 1935–42; Huene, 1942). Of these, *Prestosuchus chiniquensis* and *Prestosuchus loricatus*, occur in the Pinheiros-Chiniquá Sequence, (Middle Triassic, *Dinodontosaurus* Assemblage Zone; (Zerfass et al., 2003; Langer et al., 2007; Mastrantonio, 2010; Soares, Schultz & Horn, 2011; Horn et al., 2014). *Prestosuchus chiniquensis* and *Prestosuchus loricatus* have been considered as synonyms by Krebs (1976) and Barberena (1978), but Desojo & Rauhut (2009) argued that only the paralectotype of *P. loricatus* can be attributed to *P. chiniquensis*, while the lectotype would be of a different species. This issue was also discussed by Kischlat (2000), who attributed it to the new genus “*Abaporu loricatus*,” but this proposal is problematic because this designation only appears in the above-mentioned publication as a new generic proposal for *P. loricatus*, but this is not clearly stated in the text. As such, this proposal was not formally presented, which would be invalid according to the ICZN. Due to these problems, we consider only *Prestosuchus chiniquensis* as a valid species since it presents more referred specimens (see below).

A large skull and postcranial elements (UFRGS-PV-0156-T) were assigned to *Prestosuchus chiniquensis* by Barberena (1978); Azevedo (1991); Azevedo (1995a); Azevedo (1995b) and other authors (Sereno, 1991; Parrish, 1993; Schultz & Langer, 2007; Mastrantonio, 2010). Kischlat & Barberena (1999) proposed that it represents a new taxon, along with the paralectotype of *Prestosuchus chiniquensis* (BSPG 1933L/7). Afterwards (Kischlat, 2000) considered the more complete specimen UFRGS-PV-0152-T as belonging to this taxon, but some of the characters used in this hypothesis are problematic and have been discussed by other authors (*e.g.* Mastrantonio, 2010). Also, as in the case of “*Abaporu loricatus*,” there are nomenclatural complications.

In an abstract, Kischlat & Barberena (1999) referred to this new taxon as *Crurotarsi indeterminata*, pending an official taxonomic proposal, but in a book chapter published a year later, Kischlat (2000) already cites “*Karamuru vorax*” as a valid taxon, considered as being proposed in the above-mentioned abstract. In his doctoral thesis (Kischlat, 2003 v.1: 261) presents a “preliminary exercise in description…” of this taxon, pending a formal publication, thus the name “*Karamuru vorax*” also does not follow the ICZN parameters and must also be considered *nomen nudum*. A much more detailed discussion on this topic will be presented in a future work by other researchers (Julia Desojo, personal communication).

A mostly complete specimen (∼75%) attributed to *Prestosuchus chiniquensis* was described by Mastrantonio (2010) with a more detailed description of the braincase being published later (Mastrantonio et al., 2013). This is the most complete specimen described for this species thus far and has also added to the knowledge of possible ontogenetic variation in ‘rauisuchians’ (Mastrantonio, 2010; Mastrantonio et al., 2013; Nesbitt et al., 2013), possible paleobiological implications (Liparini, 2008; Liparini & Schultz, 2013) and phylogenetic analysis (Mastrantonio, 2010; Mastrantonio et al., 2013). Another well preserved and mostly complete specimen attributed to *P. chiniquensis* was discovered in the same locality as UFRGS-PV-0629-T, but it remains in preparation and study (Cabreira et al., 2010). The specimen UFRGS-PV-0152-T was also referred to *Prestosuchus* by Nesbitt (2011), but was never fully studied. A detailed description of this specimen will be published in a future work (Tiago Raugust, personal communication).

*Hoplitosuchus raui* was described by Huene (1935–42) and Huene (1942) as being a “rauisuchian,” but in a brief revision of this material by Desojo & Rauhut (2008) it was found to be composed by a mix of dinosaur and pseudosuchian remains and thus would be a *nomen dubium*. Previously, a right femur (BSPG AS XXV 53) and tibia (BSPG AS XXV 54) assigned to this taxon were used by Kischlat & Barberena (1999) to propose a new basal dinosaur taxon; “*Teyuwasu barbarenai.*” However, Ezcurra (2012), in a recent revision, considered *T. barbarenai* as a *nomen dubium,* due to the lack of any autapomorphies or unique combination of characters combined with the poor state of preservation of the specimen, even though it has features that are consistent with Dinosauromorpha and more inclusive clades (*i.e.* the possible presence of an anterior trochanter on the femur and the tibia with asymmetric posterior condyles on the proximal end). Huene (1935–42) and Huene (1942) also described *Procerosuchus celer,* which is considered a juvenile form of *Prestosuchus chiniquensis* (Kischlat, 2000; Desojo & Rauhut, 2009). The only recently described ‘rauisuchian’ for the *Dinodontosaurus* Assemblage Zone is *Decuriasuchus quartacolonia* (França, Ferigolo & Langer, 2011), based on 10 mostly incomplete but well preserved specimens.

In the other assemblage zones there are other ‘rauisuchian’ taxa; *Dagasuchus santacruzensis* (Lacerda, Schultz & Bertoni-Machado, 2015) was described for the *Santacruzodon* AZ (Middle Triassic; Bertoni-Machado & Holz, 2006; Soares, Schultz & Horn, 2011) and *Rauisuchus tiradentes* from the *Hyperodapedon* AZ (Late Triassic) (Huene, 1935–42; Huene, 1942; Lautenschlager & Rauhut, 2014).

In the present article, we contribute to the knowledge of ‘rauisuchians’ from the Brazilian Triassic with the description, taxonomy and phylogenetic analysis of at least two new individuals and attribute them to *Prestosuchus chiniquensis*. These fossils are from part of an assemblage that was collected in the Xiniquá (also spelled Chiniquá) region of the central Rio Grande do Sul State (Desojo, Ezcurra & Schultz, 2011). Based on comparative osteological and corroborative histological analysis (Cerda et al., 2013), these specimens contribute to the understanding of ontogenetic variation within this taxon and this variation would impact important characters that are used in cladistics studies of these forms.

### Institutional abbreviations

**BSPG**, Bayerische Staatssammlung für Paläontologie und Geologie, Munich, Germany; **CPEZ**, Paleontology Collection of the Museu Paleontológico e Arqueológico Walter Ilha, São Pedro do Sul, Brazil; **MCN**, Museu de Ciências Naturais da Fundação Zoobotânica do Rio Grande do Sul, Porto Alegre; **MCP**, Paleontology Collection of the Museu de Ciências e Tecnologia of the Pontífíca Universidade do Rio Grande do Sul, Porto Alegre, Brazil; **MCZ**, Museum of Comparative Zoology, Harvard, Cambridge, United States of America; **MSM**, Arizona Museum of Natural History, Mesa, United States of America; **NHMUK**, Natural History Museum, London, United Kingdom**; PULR**, Museu de Ciencias Naturales, Universidade Nacional de La Rioja, La Rioja, Argentina; **PVL**, Instituto Miguel Lillo, Tucuman, Argentina; **PVSJ**, Division of Vertebrate Paleontology of the Museo de Ciencias Naturales de la Universidade Nacional de San Juan, San Juan, Argentina; **SMNS**, Staatliches Museum für Naturkunde, Stuttgart, Germany; **UFRGS-PV**, Paleovertebrate Collection of the Laboratório de Paleovertebrados of the Universidade Federal do Rio Grande do Sul, Porto Alegre, Brazil; **ZPAL**, Institute of Paleobiology, Polish Academy of Science, Warsaw, Poland.

### Geological setting

The assemblage was discovered in an outcrop which belongs to the Pinheiros-Chiniquá Sequence of the Santa Maria Supersequence (Horn et al., 2014). The outcrop, like others in the general area, is composed of massive red mudstones. Biostratigraphically, it is within the *Dinodontosaurus* Assemblage Zone, which can be correlated with the Chañares fauna of the Chanãres Formation of Argentina (Rubert & Schultz, 2004; Langer et al., 2007). Chronologically, it is considered of Ladinian or Ladinian-earliest Carnian age (Desojo, Ezcurra & Schultz, 2011; Fiorelli et al., 2013; Mancuso et al., 2014).

**Figure 1:**
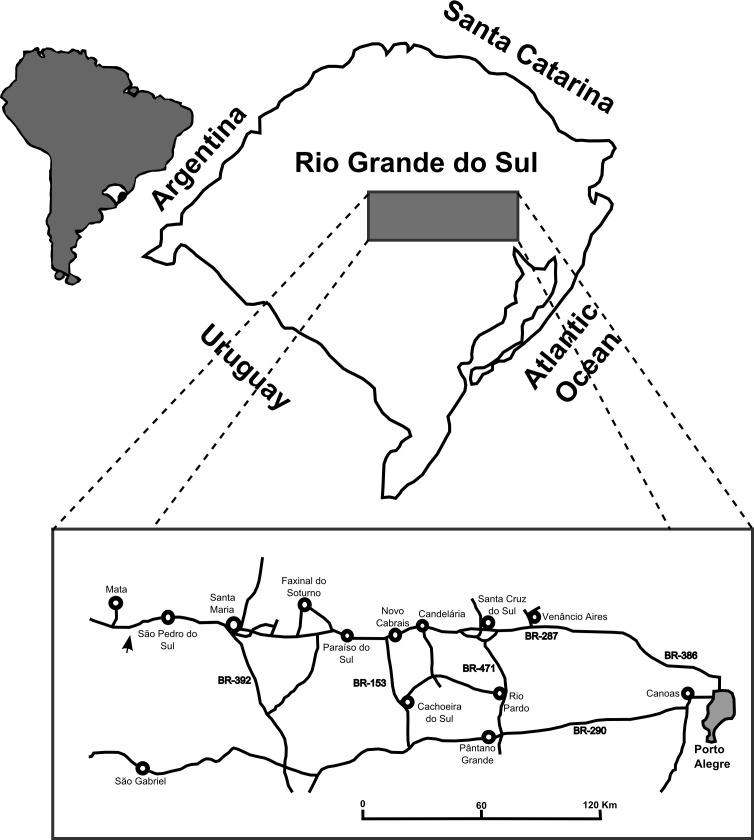
Map of the location of the “Tree Sanga” outcrop. Location of the “Tree Sanga” outcrop, indicated by the arrow (Modified from Reichel et al. (2005)).

The site is located between the cities of São Pedro do Sul, Mata and São Vicente do Sul (approximate coordinates: 29°39′21″S, 54°25′38″W (UTM: 21J 749033 6716582)), 71 km from Santa Maria (Fig. 1). This region is where Huene collected fossils in his visit to the Rio Grande do Sul State in 1928–29 and also where Stahleckeria potens (Huene, 1942), Prestosuchus chiniquensis (Huene, 1942) and Hoplitosuchus raui (Huene, 1942) were discovered. Unfortunately, the exact location of the discoveries is now lost (Desojo, Ezcurra & Schultz, 2011). This is mostly due to the loss of the reference points that Huene used in his field notes, possibly due to almost 90 years of erosion combined with growth of vegetation cover and human activities (see full discussion below).

**Figure 2:**
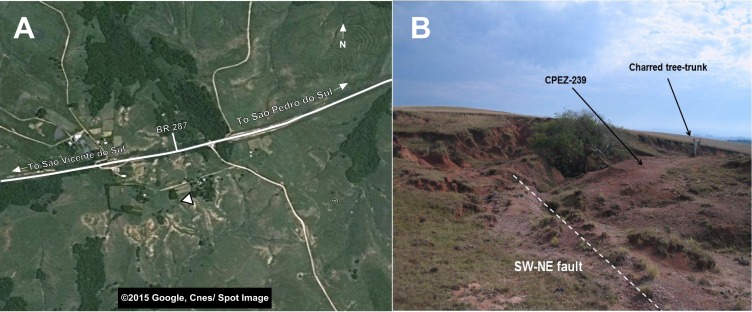
Images of the general area near the “Tree Sanga” outcrop. (A) Satelite image of the general area of the outcrop; (B) Picture of the outcrop indicating details and the area where the CPEZ-239 fossil assemblage was discovered. Map data ©2015 Google Cnes/Spot Image.

Another feature of this outcrop is the existence of a subvertical fault plane that is oriented southwest-northeast. Although it is not possible to ascertain the presence of a significant horizontal displacement between the layers on both sides of the fault, its presence would indicate that the fossils on either side of the fault might have been preserved in strata of different ages. Furthermore, the existence of nearby outcrops that display layers of approximately 5 m of vertical displacement would corroborate this condition (Fig. 2).

## Materials and Methods

Huene (1935–42) coined one of the outcrops of this region “Baum Sanga” (=Tree Sanga) due to the presence of a large “Timbaúva” tree (*Enterolobium contortisiliquum*). Beltrão (1965) reported that this tree was later struck by lightning and burned, but some of its original remains were still present at the outcrop up until the 1960’s. During the discovery of the assemblage in the early 1990’s, there were pieces of a charred tree trunk and roots close by, but with the lack of the reference points used by Huene to identify the original site, there is no certainty if this would be the its true location. A query with the owner of the private property where the original sanga and the outcrop are located, whose family lives on the land since before the time of Huene’s expedition, was unproductive (Max Langer, personal communication). As such, it is unclear if the original “Tree Sanga” and the outcrop where CPEZ-239 was discovered are the same place or are two different sites. As it is impossible to clarify this problem at the present time, we choose to follow to the designation of the site as proposed previously by Desojo, Ezcurra & Schultz (2011).

The assemblage was collected at an elevation slightly above the area near the charred tree remains. Initially, it received the designation CPEZ-239 when it was deposited in the paleontological collection of the Museu Paleontológico e Arqueológico Walter Ilha in the city of São Pedro do Sul. Desojo, Ezcurra & Schultz (2011) described the archosauriform *Archeopelta arborensis* based on part of the material and designated it as CPEZ-239a, while the rest was designated CPEZ-239b. It was never fully prepared nor described and only the complete femur was used in a comparative morphometric study (Pretto et al., 2008) and was referred as “cranial and postcranial remains of a medium-sized ‘rauisuchian’ by Desojo, Ezcurra & Schultz (2011:841). Portions of an osteoderm from a cervical vertebra were used in a histological study by Cerda et al. (2013).

The material CPEZ-239b is comprised of cranial and post-cranial elements of at least two individuals of roughly the same size, based on the presence of 2 sets of mandible bones of similar dimensions (*e.g.* articulated right maxilla with a length of 186 mm while the disarticulated right maxilla has 173 mm; complete measurements presented in the supplementary material). Of cranial elements, there is an articulated rostrum and posterior portion of a skull with associated mandibular elements and two separate jaws of a second skull. Of post-cranial material, there is a vertebral sequence including the axis, the first 8 cervical vertebrae and 3 neural arches of indeterminate position in the axial sequence. All these vertebrae display osteoderms. A right scapula and coracoid and a dorsal portion of a scapula are the only elements preserved of a shoulder girdle, while the pelvic girdle is represented by a fragment of an anterior process of an iliac blade and a ventral portion of a right ischium.

The appendicular elements are represented by a complete right humerus and an incomplete left ulna, while of the posterior elements there is a complete left femur, the distal portion of a right femur and an incomplete left tibia. The autopodials are represented only by a right fifth metatarsal and some incomplete phalanges.

The preparation of these fossils occurred in two stages: the first one was made following the fossils discovery. Along with mechanical preparation, dilute hydrochloric acid was used, but the concentration is unknown. Unfortunately, further damage to the bones occurred due to complications in the chemical preparation, hindering the clarity of some anatomical details. The fossil was originally covered by a thick layer of shellac resin, which was used as an adhesive, and the acidic reaction melted the resin which resulted in a yellowish overall hue and covering foramina, sutures and other small details in certain areas.

The second stage of preparation of this material started in 2009 by one of us (M.B.L.) as the focus of his M.Sc. dissertation, at the Laboratory of Paleovertebrados of the Universidade Federal do Rio Grande do Sul (UFRGS). At this stage, only mechanical preparation was used, where the fossils were prepared using pneumatic hammers and engravers, dental instruments, needles, hammers and chisels. Because several pieces were already fragile, some were not fully prepared so as not to risk further damage or loss. To depict these features in the schematic drawings, the grey areas indicate matrix/adhesive cover and the dark grey areas indicate damaged ones.

To facilitate the description of the more representative material (a skull with mandible), the articulated portion of the rostrum was designated “Series A,” while a posterior portion, which also features vertebrae associated with the posterior portion of a mandible was designated “Series B.” The disarticulated skull elements were not given a specific designation and have been described separately as with the rest of the material based on which body or appendage section they belong.

In the description of the cervical vertebrae, only the right side was described, because the left side was too damaged to allow visualization of structures clearly.

A comparative morphological study was made based on the observation of material at the Paleovertebrate Collection of the Laboratório de Paleovertebrados of the Universidade Federal do Rio Grande do Sul (UFRGS-PV-0156-T and UFRGS-PV-0629-T, both assigned to *Prestosuchus chiniquensis* (Azevedo, 1991; Mastrantonio, 2010)) and specimens described in the literature. The result was then tested by cladistic methodology, using TNT version 1.1 (Goloboff, Farris & Nixon, 2008). The details of this process will be discussed in the appropriate sections.

As was presented previously, CPEZ-239b consists of at least two individuals of the same species based on two pairs of morphologically similar jaw material of approximately the same size. The pattern of disarticulation of the posterior portion of a skull matches the state of the articulated rostrum (left side more disarticulated that the right one). However, it is impossible to rule out that more than two individuals are present in the assemblage, since that besides the cervical sequence that articulates with the Series B, is impossible to assign the remaining post-cranial bones at the individual level.

### Systematic paleontology

Archosauria Cope, 1869 (*sensu* Gauthier & Padian, 1985)

Pseudosuchia Zittel, 1887–1890 (*sensu* Gauthier & Padian, 1985)

Suchia Krebs, 1974 (*sensu* Benton & Clark, 1988)

Loricata Merrem, 1820 (*sensu* Nesbitt, 2011)

*Prestosuchus* Huene, 1942

*Prestosuchus chiniquensis* Huene, 1942.

Lectotype (Krebs, 1976): BSPG 1933L 1-3/5-11/28-41/41 (Excavation 34; Huene, 1942): Splenial, anterior portion of the surangular, anterior portion of the angular, prearticular, right partial maxilla, fragmentary dentary, three incomplete cervical vertebrae, fragmentary ribs, one sacral vertebrae, two sacral ribs, five anterior caudal vertebrae with chevron bones, 14 middle and posterior caudal vertebrae, right and left scapulacoracoid, interclavicle and clavicle, distal left humerus, right proximal and distal humerus, distal radius, fragmentary ulna, one manual phalanx, incomplete ilium, fragmentary ischia, pubes and a complete left hind limb.

Paralectotype: BSPG 1933L/7 (Excavation 41; Huene, 1942): a articulated vertebral sequence, composed of two sacral vertebrae with sacral ribs, incomplete last dorsal and first caudal vertebrae, dorsal portion of the right ilium, a series of osteoderms articulated with the neural spines.

Type Locality and Stratigraphic horizon: “Weg Sanga,” Pinheiros-Chiniquá Sequence of the Santa Maria Supersequence, (Ladinian), *Dinodontosaurus* Assemblage Zone.

Diagnosis: Two autapomorphies are described for this taxon (Desojo & Rauhut, 2008): an anterior notch between the scapula and the coracoid; a longitudinal ridge on the dorsal surface of the ilium. Although *P. chiniquensis* is one of the better represented ‘rauisuchian’ taxa from Brazil, only two specimens are relatively complete (see below), so the taxonomic designation of all the additional specimens was made due to overlapping characters and tested in recent phylogenetic analyses (Brusatte et al., 2010; Mastrantonio, 2010; Nesbitt, 2011; França, 2011; Mastrantonio et al., 2013). However a detailed revision of all the described specimens is needed to achieve a clear diagnosis.

### Additional specimens

UFRGS-PV-0156-T (Barberena, 1978; Azevedo, 1991): A large and complete skull, 31 vertebrae, including the axis, many with articulated osteoderms, along with unidentified fragmentary material;

UFRGS-PV-0629-T (Mastrantonio, 2010; Mastrantonio et al., 2013): A mostly complete specimen, composed of a complete but disarticulated skull, complete presacral vertebrae sequence (eight cervical, 13 dorsal), two sacral and three caudal vertebrae; complete scapular and pelvic girdle, mostly complete appendicular elements, composed of both humerus; proximal portions of the left ulna and radius; one left metacarpal of an anterior *manus*; femora, a right tibia and fibula, three isolated phalanges of a *pes*;

UFRGS-PV-0473-T (Mastrantonio et al., 2013): an isolated braincase that was attributed, with uncertainty, to *Prestosuchus chinquensis*, based on similarities between it and those of UFRGS-PV-0156-T and UFRGS-PV-0629-T;

UFRGS-PV-0152-T (Nesbitt, 2011): A mostly complete specimen. It is composed of an incomplete skull, with maxillae, nasals, quadrates, partial quadratojugal, braincase, parietal, ectopterygoid, partial pterygoid, jugal, squamosal, anterior portion of the dentary, prearticular, articular, vertebral sequence composed of cervical, dorsal, sacral and caudal elements, complete scapular and pelvic girdles, humerus, proximal portion of a ulna, femora, tibia and fibulas, complete calcaneum and *pes,* chevrons and osteoderm cover. Nesbitt (2011:33) considered this specimen to be indistinguishable from UFRGS-PV-0156-T and BSPG XXV 1-3/5-11/28-41/41, due to overlapping features, but as it is now still being fully studied (as mentioned above), we will consider Nesbitt’s (2011) interpretation of this specimen.

MCP-146 (Bonaparte, 1984): A complete pelvic girdle with the last dorsal, two sacral and three caudal vertebrae preserved in articulation. This specimen is roughly the same size as the one in UFRGS-PV-0629-T and was figured in Bonaparte (1984), but this author considered the first caudal vertebra as belong to the sacral sequence. A new inspection by one of us (M.B.L.) indicates that this might not be the case, but a detailed review will be presented in a future work.

MCZ 4167 (Parrish, 1993:297): composed of a poorly preserved but articulated specimen from the Santa Maria Formation, referable to *Prestosuchus*, but no other information is available on this specimen.

### Description

#### Series A (articulated rostrum and anterior mandibular rami)

**Figure 3:**
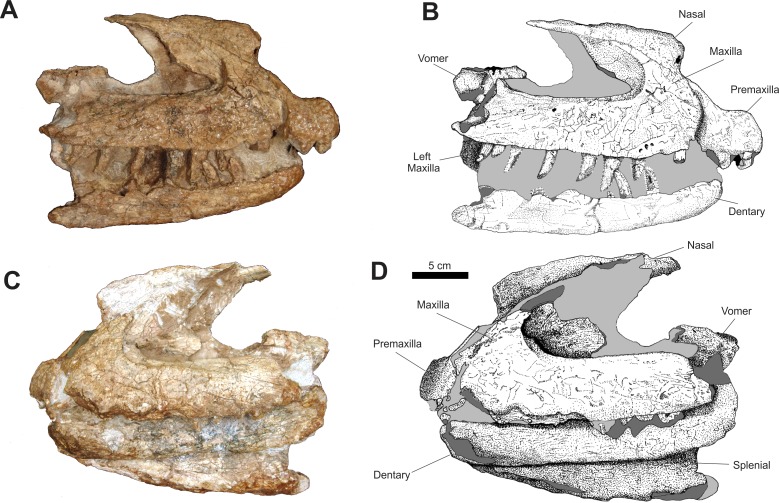
Osteology of *Prestosuchus chiniquensis* specimen CPEZ-239b, Series A, composed of a incomplete rostrum and anterior portion of a mandible. (A–B) right and (C–D) left lateral views.

In the anterior portion of the rostrum, both premaxillae are articulated, but are separated from the rest of the rostrum and laterally dislocated, causing a distortion on the anterior part of the rostrum (Fig. 3).

In lateral view, the main portion of the premaxillae is sub-rectangular, with its anterior margins slightly convex. A small depression in the anterodorsal area that, in dorsal view, displays a circular fracture that would correspond to the site of the anterior process. The anterior processes are lost, while the posterior processes are preserved, but incomplete, lacking the posterior tips. These processes are slender, similar to those in *Prestosuchus chiniquensis* (UFRGS-PV-0156-T and UFRGS-PV-0629-T; Azevedo, 1991; Mastrantonio, 2010), but not as dorso-ventrally wide as that of *Fasolasuchus tenax* (Bonaparte, 1981) and *Saurosuchus galilei* (Alcober, 2000). However, they are not as slender as those of *Decuriasuchus quartacolonia* (França, Ferigolo & Langer, 2011).

One incomplete premaxillary tooth is preserved, near a cluster of fragments of rock, bone and other teeth, which is located ventroposteriorlly to the ventral margin of the left premaxilla. On its ventral margin, the right premaxilla displays three incomplete teeth and one alveolus, which is exposed due to a fracture on the lateral surface, indicating the total count of four premaxillary teeth. This condition is described for *Saurosuchus galilei* (Reig, 1959; Sill, 1974; Alcober, 2000), *Fasolasuchus tenax* (Bonaparte, 1981), *Batrachotomus kupferzellensis* (Gower, 1999), *Postosuchus kirkpatricki* (Long & Murry, 1995; Weinbaum, 2011), *Polonosuchus* (Sulej, 2005), *Rauisuchus tiradentes* (Huene, 1935–42; Huene, 1942; Lautenschlager & Rauhut, 2014), *Prestosuchus chiniquensis* (Mastrantonio, 2010) and *Decuriasuchus quartacolonia* (França, Ferigolo & Langer, 2011).

**Figure 4:**
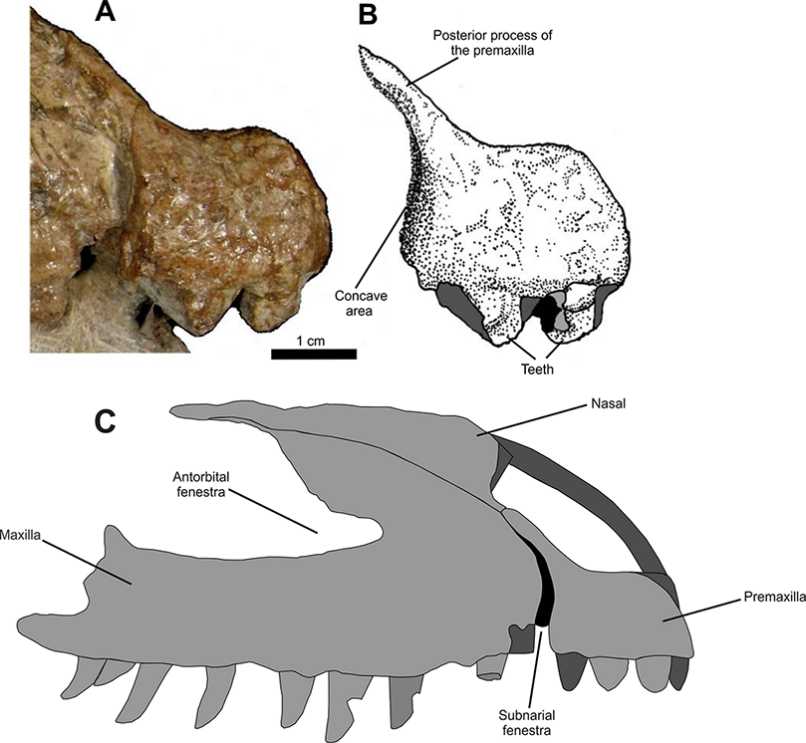
Osteology of *Prestosuchus chiniquensis* specimen CPEZ-239b, detail of the right premaxilla of Series A and its possible articulation. (A–B) indicating its posterior margin and the concave surface; (C) reconstruction of right premaxilla and maxilla, in lateral view, indicating the possible size and appearance of the subnarinal fenestra.

The posteroventral border of the right premaxilla is slightly curved anteriorly, forming a low, concave area on the posterior surface, followed by a thickened area that expands posteriorly on its posteroventral margin (Fig. 4). This concave surface is morphologically similar to the anterior margin of the subnarial fenestra described for *Saurosuchus galillei* (Alcober, 2000), *Decuriasuchus quartacolonia* (França, Ferigolo & Langer, 2011) and, in lesser form, *Postosuchus kirkpatricki* (Weinbaum, 2011). Also, it is larger than the one described for *Prestosuchus chiniquensis* (UFRGS-PV-0629-T; Mastrantonio, 2010) and while the posteroventral border of the premaxilla is similar to that of other taxa (*e.g. Rauisuchus tiradentes*; Lautenschlager & Rauhut, 2014), in other specimens of *Prestosuchus chiniquensis* (UFRGS-PV-0156-T), this area is almost vertical and doesn’t display any rough area.

In CPEZ-239b, the right maxilla of the articulated rostrum is the one that is best preserved. In lateral view, its main portion is sub-rectangular and anteroposteriorlly elongated. The ascending process is dorsoposteriorly orientated and is articulated with the ventral part of the right nasal. The dorsoposterior portion of this process is incomplete, with only an irregular dorsoventrally expanded plate of bone that forms the dorsal and anterodorsal margins of the antorbital fenestra. This area of the ascending process is located more medially than the rest of this region, boarded anteriorly by a well-marked fossa that margins the anterior to the antorbital fenestra.

The ventral border of the antorbital fenestra projects posteriorly, displaying a small ridge that precedes a small, dorsoposteriorly arranged, triangular process located on its posterior end. After this process, the border of the maxilla continues ventroposteriorly up to a fracture located above the ventral margin. The lateral surface of the posterior half of this maxilla, next to region of this fracture, is slightly lateromedially flattened. On the ventral margin, posterior to the ventral border of the premaxilla, there is a fracture that displays an empty alveolus. After this fracture, the border is damaged but displays the proximal portion of a tooth, and continues along a slight curvature, above which are three small foramina. After this curvature, the ventral margin straightens and is almost parallel to the ventral margin of the antorbital fenestra.

In lateral view, the left maxilla has its anterior margin damaged, with the area that would contact the premaxilla medially curved and with a smooth surface. There is a sinuous fracture that exposes small tooth fragments. After this fracture, this margin continues straight until its posterior portion displaying three incomplete teeth.

On its dorsal margin, the ascending process is incomplete, with only its ventral portion preserved. The sheet of bone that would correspond to the medial portion of the antorbital fossa is dorsoventrally expanded and only the area closest to the anterior tip of the antorbital fenestra is preserved. The left antorbital fossa is deeper than its right counterpart, but this condition is possibly due to diagenetic factors, since the left maxilla is more laterally expanded than the right one.

The shape of the anterodorsal border of the maxilla and its proximity to the antorbital fenestra is very similar to that found in *Prestosuchus chiniquensis* (UFRGS-PV-0156-T, UFRGS-PV-0629-T), *Teratosaurus suevicus* (NHM 38646), *Polonosuchus silesiacus* (ZPAL AbIII/563) and *Batrachotomus kupferzellensis* (SMNS 80260), which are comparatively anteroposteriorly shortened, whereas this same region in *Fasolasuchus tenax* (PVL 3851) and in a lesser condition in *Saurosuchus galilei* (PVL 2062; PVSJ 32) tends to be more elongated posteriorly. The overall aspect of the ascending process and the fossa of the antorbital fenestra is very similar to that present in *Prestosuchus chiniquensis* (UFRGS-PV-0156-T and UFRGS-PV-0629-T). In specimen UFRGS-PV-0156-T both suffered diagenetic alteration.

The two nasals are preserved in the articulated rostrum of the Series A. Both bones are incomplete, with only the medial portion closest to the contact of both nasals being preserved. In lateral view, the right nasal is articulated with the ascending process of the right maxilla and with the left nasal. Its descending process is medial to the ascending process of the premaxilla due to the dislocation of the latter.

The anterior portion of the right nasal is laterally compressed. This area is almost flat and is projected anterodorsally, exposing a part of the inner channel of the nasal and its posterior portion is convex. Above this area, the margin of the nasal is orientated dorsally until it is parallel to the sagittal axis, where it follows posteriorly, with a slight concave area on half its body. A small laterally projected ridge is present and follows until the end of the preserved bone. A portion of the posterior length of the nasal is visible posteriorly to the lateromedially compressed area.

The nasals are similar to those of *Saurosuchus galilei* (Alcober, 2000) and of *Prestosuchus chiniquensis* (UFRGS-PV-0156-T and UFRGS-PV-0629-T). A rugose area present along the dorsoventral margin of this bone is described for *Batrachotomus* and *Postosuchus*, while a more reduced one is described for *Prestosuchus* and *Rauisuchus*. Furthermore, in the former taxon, a depression on the dorsal area of the skull is similar to the one present on the nasals of CPEZ-239b.

In lateral view, the convex aspect of the nasals appears to form a roman nose aspect that is described in some taxa. However, this is not the case in CPEZ-239b, since both bones were laterally compressed, which clearly caused a dorsoventral expansion of the nasals that gave them the aforementioned aspect.

Associated to the dorsoposterior portion of the maxilla and located medially in this series are two incomplete vomers. In lateral view, their form is of an irregular lateromedially compressed bones that are dorsally dislocated. Their anterior portion is absent and the posterior portion displays a small depression, limited anteroventrally by two small, damaged and incomplete ridges. On the ventral portion of the left vomer there is an incomplete process that is oval in shape, and is slightly lateromedially flattened, which is located ventrally to its anterior portion. This appears to be a border of a fenestra, but due to the incomplete nature of this bone, it is impossible to correctly determine.

Of the incomplete jaw, the right mandibular ramus displays an anteroposteriorlly elongated dentary, which is slightly curved anteriorly, with a short dorsoventral convex expansion on its anterior tip in lateral view. On its ventral margin, there are five incomplete teeth while the rest of the alveoli are not visible. In lateral view, the left mandibular ramus is more dorsally placed than its right counterpart, located almost between the right and left maxillae. In this view, only the dentary is present. This bone is fractured on its anterior edge and displays a posterior margin that is more complete that the right one, but due to damage by chemical preparation, it is impossible to identify sutures or if there are any other parts of articulated jaw bones. In left lateral view, the right splenial is visible and articulated on the medial side of the right mandibular ramus. The dorsal portion of this bone is hidden due to the obstruction of the left mandibular ramus, but it appears that its overall aspect is of a sub-triangular bone, with its tip orientated anteriorly and its ventral margin following obliquely the ventral border of the dentary, until near the posterior border of the latter, where it projects dorsally by a sinuous suture along a fractured area.

#### Series B (posterior portion of a skull and mandibles)

**Figure 5:**
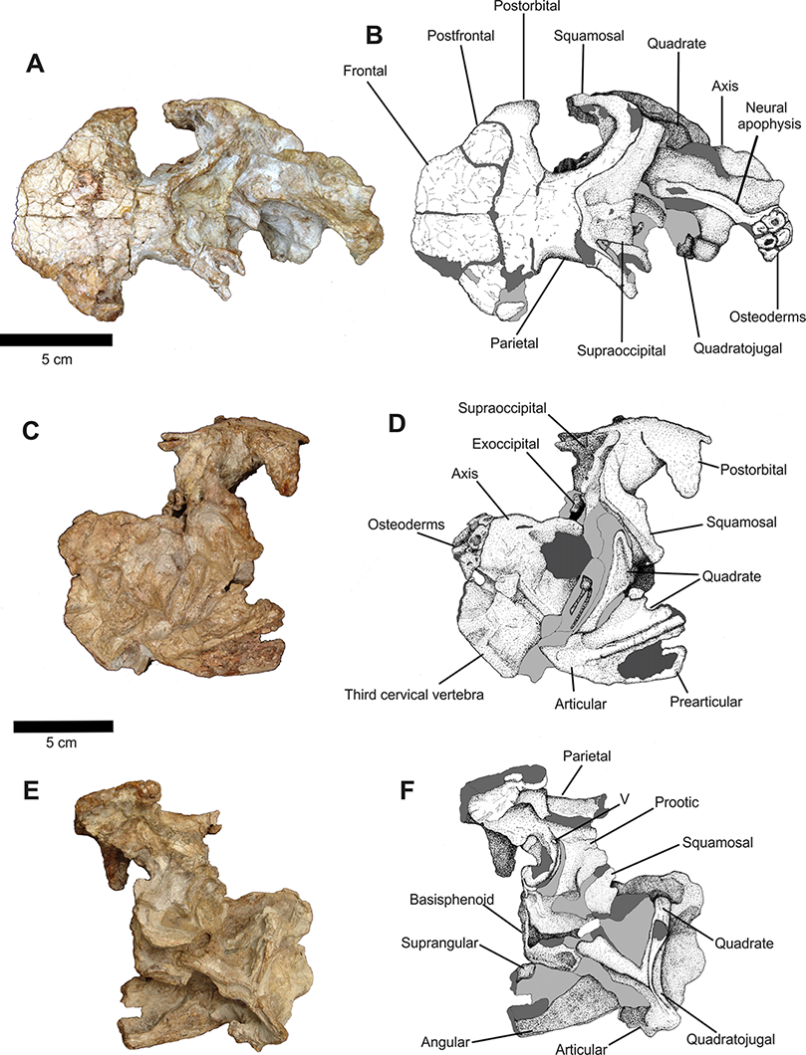
Osteology of *Prestosuchus chiniquensis* specimen CPEZ-239b, Series B composed of dermal roof, neurocranium and mandibular elements. (A–B) dorsal, (C–D) right lateral and (E–F) left lateroventral views.

The majority of the sutures of the dermal bones in this series have been eroded and the contact between the bones has been widened (Fig. 5). However, these sutures appear to not have been completely fused initially. The anterior end of the skull roof is formed by the posterior portions of two incomplete frontals, with the right one being better preserved, while the left one displays a large fracture on its lateral portion. They are sub-rectangular, dorsoventrally compressed bones that meet lateroposteriorly with the dorsomedial border of the postfrontal. Posteriorly, a large suture (that clearly has been widened due to preservation) delimitates the articulation with the postfrontals and with the anterior margin of the parietals.

The postfrontals are small, circular in dorsolateral view, dorsoventrally flattened bones that form, along with the lateral border of the frontals and the anterior margin of the postorbitals and the dorsoposterior border of the orbit. This detail is better preserved on the right side of the skull.

The right postorbital is better preserved than its left counterpart. In dorsal view, it is articulated ventrolaterally with the postfrontal, which projects laterally to form, a ventrally projected process that would form the ventroposterior margin of the orbit which is more clearly visible in lateral view. Of the left postorbital, only the portion close to the articulation with the other bones of the skull roof is preserved.

It is unclear if the parietals are fused since the corresponding area is badly damaged. Although there are fractures running parallel to the sagittal axis of this element(s), we describe the parietal as a single bone. It is located on the third half of this part of the skull. In dorsal view, the parietal is sub-rectangular, laterally convex, with its anterior portion expanded anterolaterally, articulating with the posterior margin of the frontal and the postorbital along the widened suture. From its middle half, two posterior processes project posterolaterally, with the right one being better preserved than its left counterpart, and delimitating the posterior margin of the skull with the occipital region and forming the dorsal and dorsoposterior border of the supratemporal fenestra. There is no evidence of a sagittal ridge, condition similar to what is found in *Prestosuchus chiniquensis* (Mastrantonio, 2010) and *Saurosuchus galilei* (Alcober, 2000).

The overall aspect of the right supratemporal fenestra is less damaged than the left one. Both the interior portions of the fenestra are smooth, with no indication of a ridge or any other structure. On the lateral margin of the parietal, located medially to the supratemporal fenestra, there is a small ridge that limits both fenestra, but this structure is clearly due to a fracture on both the lateral sides of the parietal.

In right lateral view, the dorsoposterior border of the orbit curves lateroventrally, forming a slender, ventrally projected process that is composed of part of the postfrontal and of the postorbital, that is dorsoventrally elongated and has a damaged rounded tip.

The posterior portion of the parietal displays a slight ventral curvature. In lateral view, it is possible to see that the body of this element forms all the dorsolateral wall of the dorsal region of the neurocranium. It is not possible, in this view, to establish the presence of a suture between the parietal and the dorsal elements of the skull due to preservation.

The right squamosal is a lateromedially compressed bar that is positioned ventrally, starting from its suture with the parietal and ending in a rounded area, which marks the dorsoposterior margin of the infratemporal fenestra, while also being projected slightly anteriorly. There is no evidence of an anterior process (the “stepped” middle portion *sensu* (Brusatte et al., 2010), that would be an anterior projection of the squamosal in the infratemporal fenestra) on the anterior margin of the squamosal.

Located posteriorly to the squamosal there is an incomplete ramus of the right quadratojugal. It is dislocated posterolaterally and is more medially displaced than the squamosal, close to the lateral area of the occipital region. A ventral portion of the quadratojugal is near to the dorsal margin of the portion of the right mandibular ramus that is associated in this series.

In right lateral view, the prootic is present, but its exact form cannot be determined and its contact with other elements is not clear. Anteroventrally, its anterior margin meets the laterosphenoid and forms the posterior border of the trigeminal foramen. The area circumventing this foramen on the right side is expanded and damaged. Ventroposteriorlly to the prootic, a large circular, indeterminate fragment is located, with a concave surface on its dorsolateral area. This fragment covers the right lateral side of the neurocranium, along with a concretion composed of fragments of sediment and bones. Due to the damaged condition already discussed, this area was not further prepared.

Two incomplete quadrate parts are preserved. The first one is the dorsal portion that is associated to the lateral portion of the quadratojugal. The other part is the ventral third of this element that is still articulated with the posterior portion of the right mandible. This portion of the mandible is dislocated posterodorsally and is positioned dorsally on the posterior portion of the skull.

The majorities of the dermal bones on the left side of the posterior part of the skull area are disarticulated or absent, thus exposing the neurocranium in lateral view. The absence of sutures appears to indicate that an incomplete left prootic occupies all the dorsal portion of the neurocranium, ventrally to the parietal and separated by a fracture. Its anterior margin is sinuous and follows posteroventrally to a lateral fossa, where the trigeminal foramen is located, close to a large fracture. This area continues deeply from its anterior border and curves anteriorly, forming a similar structure to a channel that would be the area of the eustachian tube. Furthermore, this area was distorted to the left at an approximately 45° angle.

On its lateral body, the left prootic is bordered ventrally by an area of concretion. Dorsal to this, there is an incomplete portion of the prootic ridge, highlighted from the rest of the prootic because it is laterally expanded and with an oval cavity located ventrally. The overall shape of this area and the position of the foramen are almost identical to the one on the right side of the skull and, aside from the trigeminal foramen, there is no evidence of foramina on this bone.

The basisphenoid is split in two halves that are dislocated along the sagittal plane, with the left halve being oriented more dorsally while the right one is located more ventrally. Both halves are dislocated laterally, following the deformation of the anterior portion of the neurocranium. The ventral portion of the right one is visible and is sub-rectangular in form, with a shallow fossa on its posterior half. A fragment of the dorsal portion of the left squamosal is preserved but is dislocated ventrally near an opening formed by an extension composed of part of a concretion and the posterior portion of the basisphenoid, leaving only the lateroposterior portion of the left squamosal visible.

The quadratojugal is divided into two parts. Its dorsal portion is adhered to a concretion and is laterally dislocated, positioned ventrolaterally to the foramen magnum. The other portion is located near the posterior of Series B, lateral to the associated cervical vertebrae. The form of the quadratojugal is of a tri-radiated bone that has an anterior process projected medially, with its anterior tip located close to the medial region of the left portion of the basisphenoid. Another process is oriented dorsally and located laterally to the left quadrate. It follows posteriorly the main body of the quadratojugal along a posterior process of the left quadrate that extends a little short of ventral area of articulation of this bone. A fragment of the medial face of the quadratojugal, arranged more medially, is articulated with the articular surface of the quadrate. Their overall aspect is similar to the ones found in *Batrachotomus* (SMNS 52970 and 80260), *Saurosuchus* (PVSJ 32) and *Prestosuchus* (UFRGS-PV-0156-T and UFRGS-PV-0629-T) with the position of the quadratojugal and the jugal being similar to the one in *Prestosuchus*, where the quadratojugal in lateral view is more exposed than the jugal.

The quadrate, in lateral view, is articulated with the quadratojugal, but is positioned more medially among the dorsal and posterior processes of this bone and is located close to the lateral area of the cervical vertebrae. Its aspect is of anteroposteriorly elongated bone, with a slightly convex body that ends in a concave, lateromedially expanded, articular condyle.

A small fragment, anteriorly orientated, and articulated with the medial border of the quadrate and lateroventrally to the third cervical vertebra, is possibly a piece of the medial portion of the articular of the left mandible.

**Figure 6:**
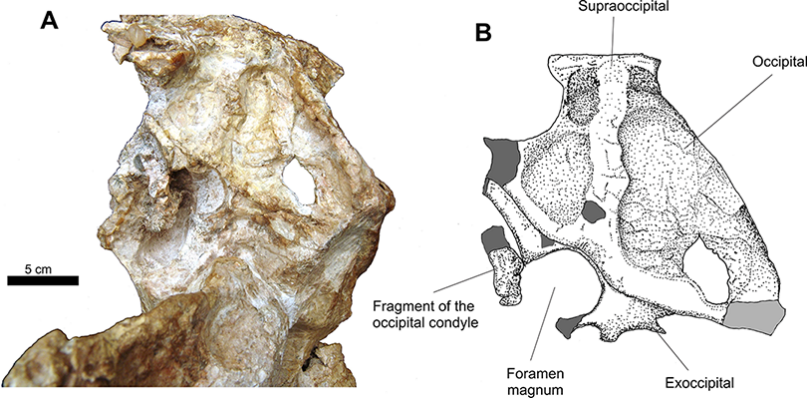
Osteology of *Prestosuchus chiniquensis* specimen CPEZ-239b, detail of Series B in occipital view. (A) close up picture of the cranial elements of Series B in occipital view; (B) close up illustration of the area around the foramen magnum.

The occipital region is incomplete (Fig. 6). There are two fragments of the exoccipital that border the foramen magnum and two other elements that are the dorsoposterior portion of the basioccipital. Due to diagenetic alteration, this region was pushed anteriorly, which resulted in the posterior portion of the neurocranium being anteroventrally inserted in the skull cavity while another dislocation further altered this area in the horizontal plane. This is inferred based on the odd arrangement of the occipitals relative to the rest of the skull and the absence of the lateral portions of the exoccipitals, the paraoccipital process of the opisthotics and the uneven level of the suture of the supraoccipital with the exoccipitals.

The supraoccipital is sub-triangular, meeting dorsally with the posterior margin of the parietal and directed ventrally with lateral expansions, until it is articulated with the occipitals, similar to the ones found in *Batrachotomus* (SMNS 80260), *Prestosuchus* (UFRGS-PV-0156-T), *Saurosuchus* (PVSJ 32) and *Arizonasaurus* (MSM P4590). The left lateral and ventral border of this bone are distorted due to compression, which resulted in the left side of this element being comparatively smaller than its right counterpart. A crooked, low dorsal ridge is present. On both sides of this ridge there are shallow fossae.

The exoccipitals form the lateroposterior border of the foramen magnum and are articulated with the occipital dorsally along the ventral margin of this bone. The posterior process of the left exoccipital is more complete than its right counterpart, with a fragment of the occipital condyle of the basioccipital preserved.

Associated with this series is an incomplete posterior portion of a right mandibular ramus. In right lateral view, it is impossible to identify the sutures of the bones that would compose this structure. This portion of the mandibular ramus is anteroposteriorly elongated, with its ventral border curving anteroposteriorly up to the posterior margin of the articular. Ventrally to this contact there is there is a well-defined ridge that is parallel to the dorsal margin and continuously reduces in height up to the lateroposterior face of the articular.

In left lateral view, only the posterior portion of the prearticular and the medial portion of the surangular are preserved. The prearticular is expanded anteroposteriorlly, with its dorsal border parallel to its ventral margin, until a dorsoposterior border. The suprangular is a mostly flat bone, which displays a depression on its medial surface.

### Disarticulated skull elements

**Figure 7:**
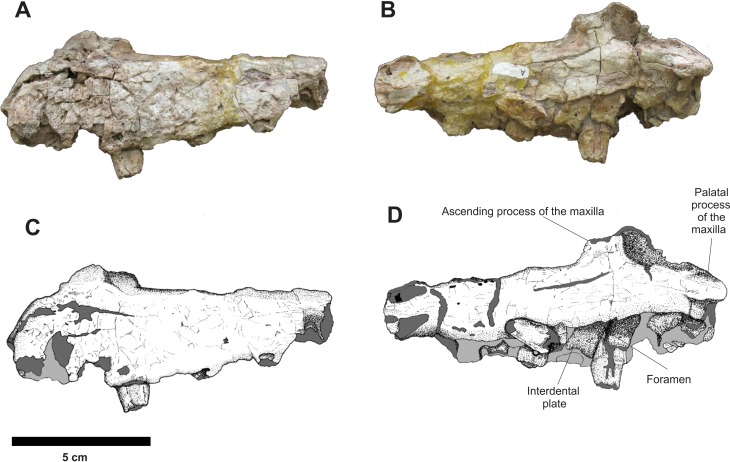
Osteology of *Prestosuchus chiniquensis* specimen CPEZ-239b, left desarticulated maxilla. (A–C) lateral and (B–D) medial views.

**Figure 8:**
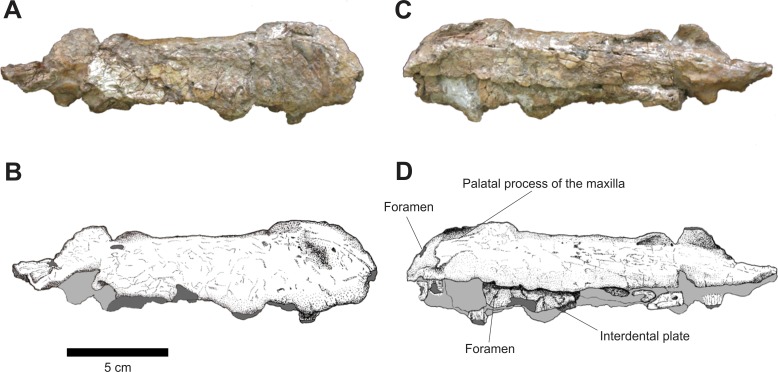
Osteology of *Prestosuchus chiniquensis* specimen CPEZ-239b, desarticulated right maxilla. (A–B) lateral and (C–D) medial views.

The left and right isolated maxillae do not display any great morphological differences in comparison to the maxillae present in the Series A. The most notable variation between these two isolated elements is that, in lateral view, the right maxilla is more anteroposteriorly elongated while the left one is comparatively shorter and its anterior portion is more dorsoventrally expanded. The preserved incomplete teeth are similar to those found in the articulated rostrum, but their condition is poor and it is impossible to determine the number of alveoli on each maxilla. In medial view, both maxillae display articulated, slightly sub-rectangular dental plates. On their medial side, these plates are perforated by small foramina (Figs. 7 and 8).

**Figure 9:**
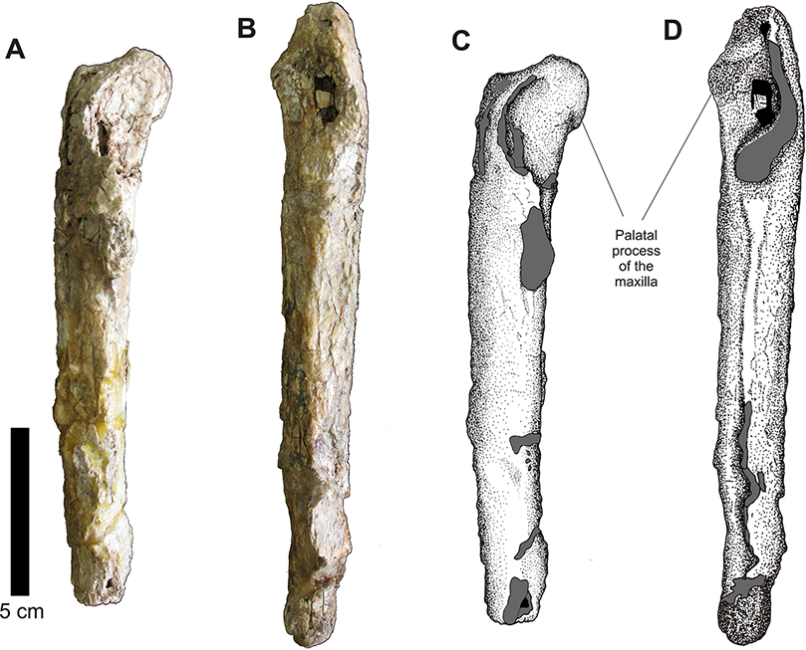
Osteology of *Prestosuchus chiniquensis* specimen CPEZ-239b, desarticulated maxillae. (A–C) left and (B–D) right disarticulated maxillae in dorsal views.

In dorsal view, the right disarticulated maxilla is elongated and lateromedially compressed. Its anterior portion displays a medial orientated curvature that forms the palatal process (= anteromedial process; Galton, 1985). This process extends beyond the anterior border of the maxilla. On its anterior face, there exists a small opening that would be for the passage of a rostrolateral/anterior opening foramen. This structure is better preserved on the left maxilla, because the same area on the right one has a fracture that begins on the most anterior margin and continues posterolaterally along the lateral surface of this bone (Fig. 9).

The presence of only one lateral anterior foramen is described in suchian taxa for *Teratosaurus suevicus* (Galton, 1985). *Batrachotomus kuperferzellensis* (Gower, 1999), *Polonosuchus silesiacus* (Sulej, 2005) and *Prestosuchus chiniquensis* (Mastrantonio, 2010). In CPEZ-239b, both disarticulated maxilla display a small opening in the general region where this foramen occurs in other taxa, but their position varies slightly in both elements due to diagenetic alteration of the dimensions of the bones.

**Figure 10:**
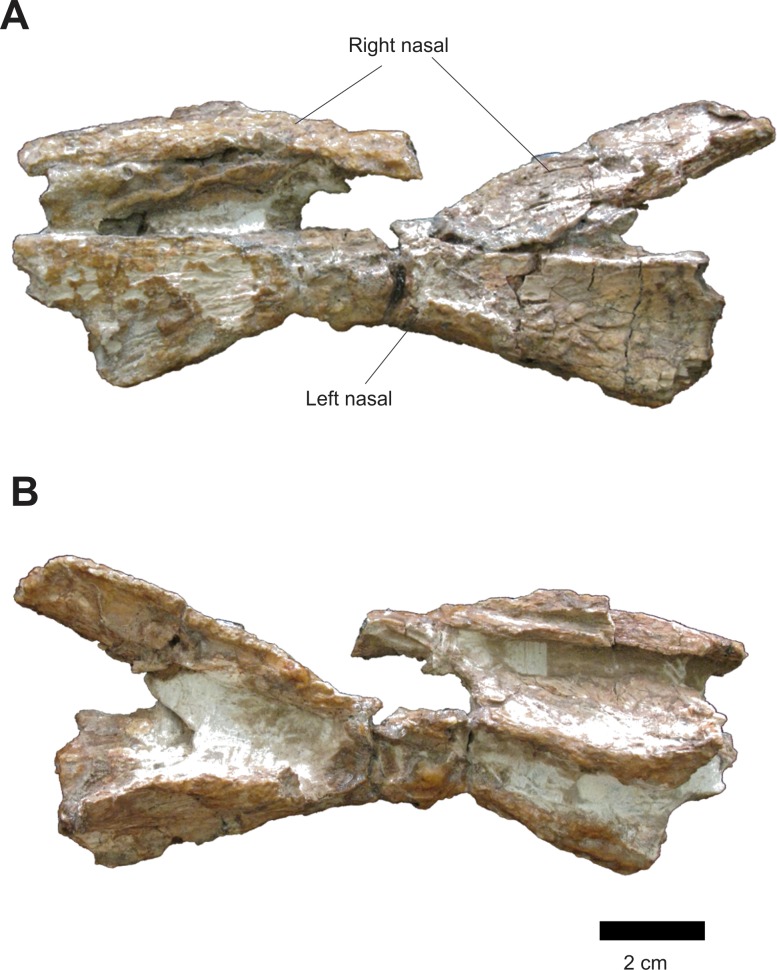
Osteology of *Prestosuchus chiniquensis* specimen CPEZ-239b, left and right disarticulated nasals. (A) dorsal and (B) ventral views.

Both isolated nasals are in articulation (Fig. 10). The left nasal has its anterior portion orientated horizontally while its posterior portion is projected anteroposteriorly. Anteriorly, on the left nasal, there is a preserved portion of the anterodorsal process and the posterior margin of the external naris. A fracture along the rugose area on the laterodorsal face of this nasal caused its posterior portion to be split in two. Apparently, the right nasal suffered a compression, leaving it with a concave form and its lateral border directed medially.

In medial view, there is a sheet of bone that is similar to the one that separates the nasal septum, but in this case, it is likely to be medial portion of a nasal that is distorted and turned ventrally. The left nasal is compressed laterally, being represented only by the portion that would contact the right nasal, in lateral view, the compression of the left nasal is more apparent. The form of the nasal is overall similar to those of the Series A. Furthermore, the left nasal displays a shallow depression on its posterior portion that would be similar to that present on the right nasal of the Series A.

**Figure 11:**
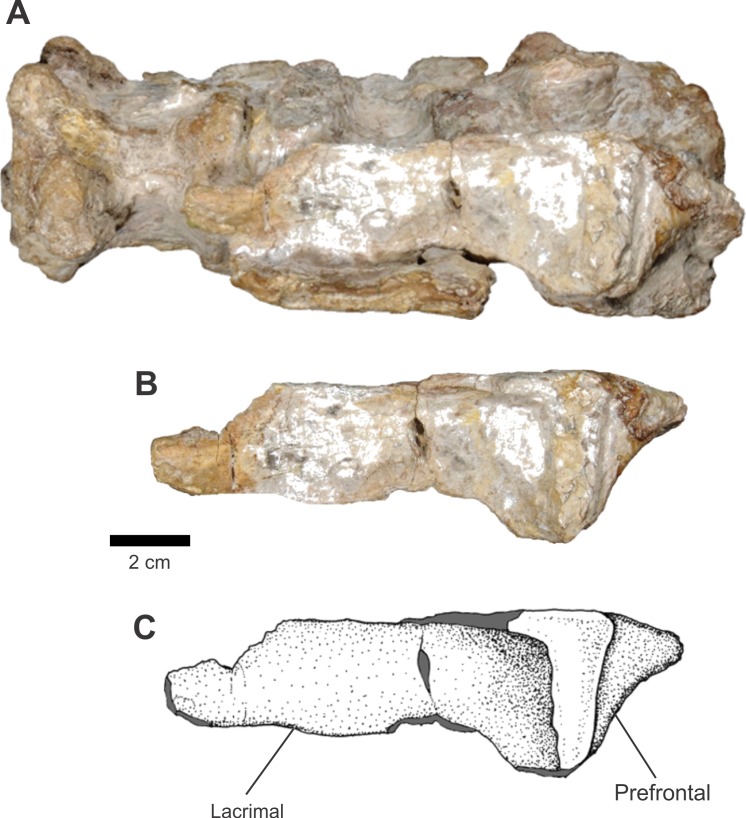
Osteology of *Prestosuchus chiniquensis* specimen CPEZ-239b, left lacrimal and prefrontal. (A) ventral portion of the cervical vertebrae; (B) close-up and (C) schematic illustration of the left lacrimal and prefrontal in lateral views.

There is only one articulated left lacrimal and prefrontal in this assemblage of bones. They are associated to the ventral portion of a cervical vertebrate sequence (Fig. 11). In lateral view, the lacrimal is rectangular, with its posterior portion slightly expanded dorsoventrally and the anterior portion is incomplete. Dorsoposteriorly, there occurs a thickening, forming a ridge that extends up to the end of the dorsal margin and continues ventrally along the posterior border. On the end of this ridge, located ventroposteriorly, there exists a small structure that would be the dorsal margin of the lacrimal canal.

The anterior portion of the prefrontal is triangular shaped, with its vertices posteriorly directed. This point is a small process that projects posteriorly and whose tip is absent. Its surface of articulation with the lacrimal is a wide area that occupies all the anterior margin of the body of the prefrontal.

Of the isolated mandibular elements, there are two damaged, incomplete left and right rami. The right one is better preserved than its left counterpart. Also, the left one is more lateromedially expanded, that probably caused its break and distortion, as well as the alteration in the orientation of its dorsal margin. This alteration makes both fragments seem to be of the same side, but the distortion of the dorsal margin is clear and there are pieces of the left splenial on its medial side.

The right mandible, in dorsal view, is elongated and compressed lateromedially. It also displays a medial pointing distortion that is more pronounced on its anterior and posterior sides. Due to its preservation, it’s possible that this distortion was heightened by diagenetic action, especially on its posterior region. There are five incomplete teeth and some alveolus are present in this ramus, but its dorsal border displays large concretions that makes it impossible to establish the exact number of teeth.

**Figure 12:**
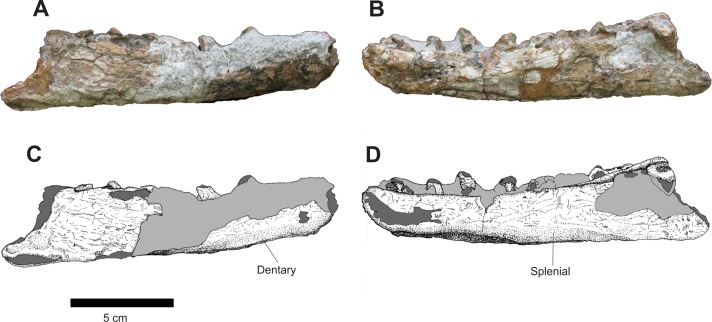
Osteology of *Prestosuchus chiniquensis* specimen CPEZ-239b, desarticulated right mandibular ramus. (A–C) lateral and (B–D) medial views.

In medial view (Fig. 12), the area of articulation of the mandibular symphysis of the right incomplete mandible is broken. On its dorsal margin, there are a series of incomplete teeth and the dorsal margin of fractured dental plates. The splenial is an elongated bone. Its anterior portion contacts the posterior margin of the approximated area of the contact with the mandibular symphysis by a long, anterior process, which follows dorsally up to the border of the dentary. The ventral margin follows the length of the ventromedial surface of this bone, while its dorsal margin is too damaged to establish its limits.

**Figure 13:**
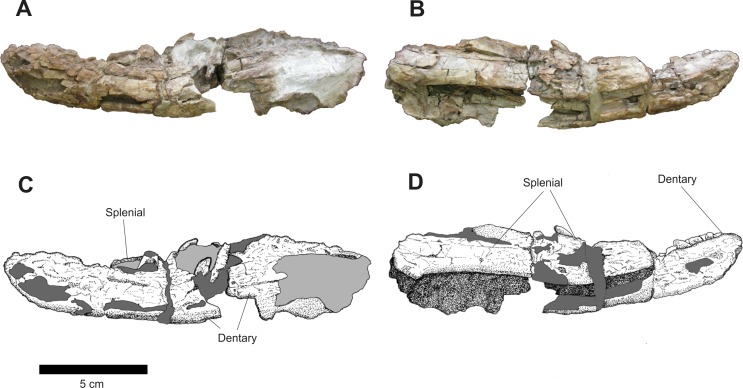
Osteology of *Prestosuchus chiniquensis* specimen CPEZ-239b, desarticulated left mandibular ramus. (A–C) lateral and (B–D) medial views.

The left mandible is the most damaged (Fig. 13). A large fracture on its posterior half shows a mesial located area that would be the space for the meckelian channel. In medial view, it is similar to its right counterpart, but possibly due to the fracture, the dentary is more laterally displaced while the splenial is comparatively located more medially.

Of the disarticulated mandibular rami, the right one displays eight incomplete teeth and an undetermined number of alveoli due to concretions. Furthermore, there is no evidence of dental plates. On the left one, there are six incomplete teeth. The more ventrally located tooth is exposed, laterally, due to the damaged state of this ramus, and is still located inside its alveolus.

**Figure 14:**
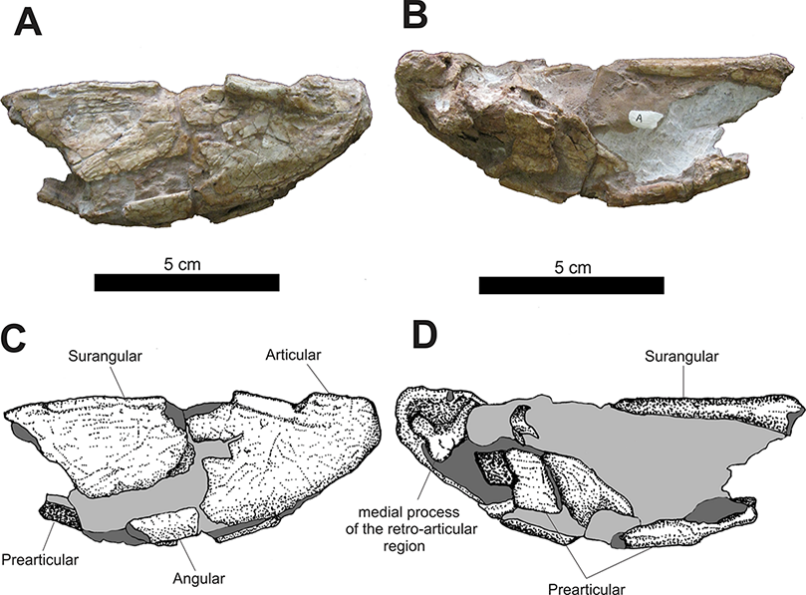
Osteology of *Prestosuchus chiniquensis* specimen CPEZ-239b, posterior portion of a left mandibular ramus. (A–C) lateral and (B–D) medial views.

An incomplete posterior portion of the left mandible is preserved (Fig. 14). In lateral view, the anterior portion of this fragment is formed by the posterior margin of an incomplete surangular located anterodorsally with the anterolateral portion of the prearticular located ventrally. The articular is preserved and occupies the area of this fragment. Its shape is sub-triangular, with a rounded area of articulation corresponding to its apex directed posteriorly. At the dorsal edge of the articular, after a fracture located on the edge of its posterior margin, there is a slightly perpendicular, laterally projected crest, whose height continuously decreases up to near the posterior portion of the articular.

In medial view, a large concretion occupies the entire medial portion of this fragment. The medial aspect of the supraoccipital occupies the anterodorsal portion of this ramus, while incomplete fragments of the prearticular occupy the ventral region of the posterior fragment of the left mandible. In the posterior portion, located after a damaged area and a concretion is the process of the medial region of the retro-articular joint. This process forms a basis for a dorsoventral fossa, located posteriorly on the medial side of the joint.

### Postcranial elements

**Figure 15:**
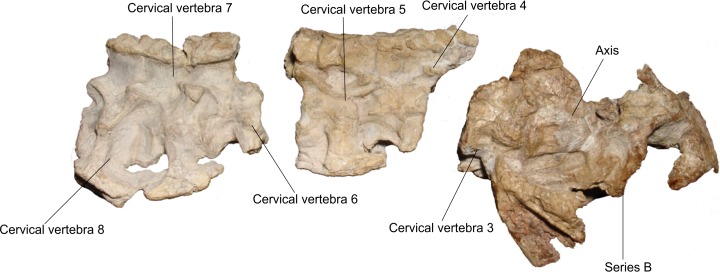
Osteology of *Prestosuchus chiniquensis* specimen CPEZ-239b, cervical sequence. Arranged cervical sequence of CPEZ-239b in left lateral view.

Of the axial skeleton, there are seven cervical vertebrae, separated into three sets (Fig. 15) and three incomplete vertebrae represented only by parts of the neural arches, not being possible to infer their place in the vertebral series (Fig. 17).

The cervical vertebrae, with the exception of the axis and the third cervical vertebra, have only the right side preserved enough to identify their structures, while the left is severely damaged by acid corrosion. Additionally there are four incomplete cervical ribs, preserved in articulation with on vertebrae Cv5, Cv7 and Cv8 and only the dorsal portion of the rib preserved in Cv6.

The three series of cervical vertebrae consist of: a set formed by the axis and the third cervical vertebra (Cv3) that are associated with the posterior portion of the cranium described as Series B. Another set is represented only of the dorsal neural apophysis and the third vertebra osteoderms, in articulation with the fourth and fifth vertebrae (Cv4 and Cv5) and the final set composed of the sixth, seventh and eighth cervical vertebrae (Cv6, Cv7 and Cv8). There is no evidence of the atlas because all elements ventral to the foramen magnum were lost.

In the anterior end of the first cervical sequence, the right side displays a large carbonate concretion that covers the entire anterior portion of the axis, leaving only its spinal apophysis and the posterior half of its centrum exposed. On the axis there is a small pit, located laterally next to a fracture that is in the anterior portion of the vertebra. Its right postzygapophysis was fractured along with the prezygapophysis of vertebra Cv3 due to a distortion that displaced the latter vertebrae more dorsally. On the axis there is also a thickening on the posterior margin of the vertebral center. The neural spine of the axis, in lateral view, is a sub-triangular, lateromedially compressed blade that is posterodorsally projected, with a slightly convex dorsal margin and an irregular border, culminating dorsally with a few small fragments of osteoderms on its dorsal tip.

The Cv3 is the only vertebra in which the posterior region is exposed and relatively undamaged. The opening of the neural channel is tear-shaped with the tip tapering dorsally. This is due to a fracture that exposed the posterior part of the neural arch. The channel is filled with sediment and the opening is covered by a thick layer of coating, which prevented further preparation by the fear of further damage this structure.

**Figure 16:**
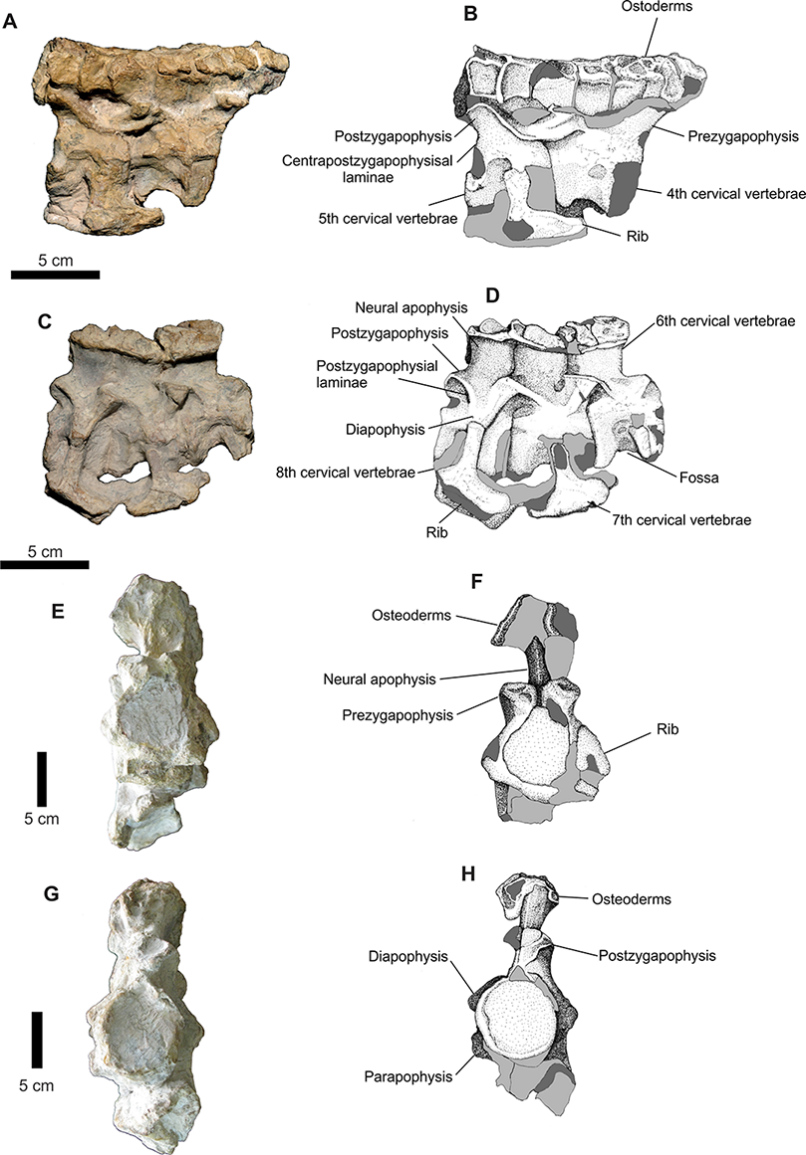
Osteology of *Prestosuchus chiniquensis* specimen CPEZ-239b, cervical vertebrae. (A–D) first and second sets of cervical vertebrae in right lateral views; (E) second set of cervical vertebrae and (F) schematic drawing of only the 6^th^ cervical vertebrae in anterior view; (G) second set of cervical vertebrae and (H) the 8^th^ cervical vertebrae in posterior view.

The second set of vertebrae, formed by the vertebrae Cv4 and Cv5, presents, in lateral view a projection on its anterior edge which corresponds to the rounded portion of the neural spine of the vertebra Cv3, which is present in the assembly described above (Fig. 16). The row of osteoderms of this series is arranged more laterally than the second set, particularly in its most anterior third. The neural spine of Cv4 is covered by a layer of concretions, while only the rear portion of the Cv5 is visible. The dorsal margin of the Cv5 vertebra is prominent, especially its area near the postzygapophyses. One area covered in concretions occupies the entire ventral region of this vertebra. A rib is articulated with the Cv5 vertebra, but the aforementioned concretion prevents a clear view of its articulation with the vertebrae. Only the proximal part is preserved. Its anterior portion is anteroposteriorly elongated; with the posterior sub-triangular process of rib is thinning posteriorly.

The third set of cervical vertebrae is better preserved than the previous ones (Fig. 16). The centrum is equilateral. The Cv6 vertebra has a fossa on its ventrolateral portion. In ventral view, there exists a small depression that is parallel to the sagittal axis. This depression appears to be bordered by two small ridges that run parallel the inside edge of a joint surface from the center to the other. The other two vertebrae have few areas exposed. The neural spine of these vertebrae is also visible. In side view, they are short and are anteroposteriorly wide.

The prezygapophysis-postzygapophysis laminae are more apparent in the Cv8 while the blade that connects the center of the vertebra with the zygapophyses is better preserved than in Cv5 in Cv8, since this last it was eroded this blade.

The parapophysis is best preserved in the Cv8 and is positioned anterioventrally in the vertebral body, with the diapophysis as a short transverse process. The transverse process and its articulation with the tubercle of a proximal fragment of a rib are best preserved in Cv7 vertebrae. The overall aspect of the ribs is more apparent in the one articulated with vertebrae Cv5, although its anterior edge is more rounded.

The cervical vertebrae are similar to the ones in other ‘rauisuchian’ taxa, with a shortened, slightly amphycoelic vertebral centra, with two surfaces for articulation and an anteroposteriorly widerneurapophysis, when compared with the centra. This pattern is matches the one found in *Prestosuchus* and *Stagonosuchus*, but differs from the one in *Batrachotomus*, *Fasolasuchus* and *Rauisuchus*.

Three other incomplete neural arches are preserved. These display two parallel rows of osteoderms articulated with the neural apophysis of two vertebrae. The shape of these osteoderms is similar to those found in cervical sequence described above, but these are much more damaged and no structure is apparent on its dorsal surface. It is impossible to determine which portion of the vertebrae they belong to, due to their damaged state, although there size is similar to those of the cervical vertebrae.

All osteoderms present in CPEZ-239b are in articulation with their corresponding vertebrae. They increase gradually in size along the axial skeleton, with the first ones as small irregular discs with a central fossa and the posterior ones being larger, slightly oval shaped, with a sinuous posterior border and a dorsal ridge, such as the better preserved ones articulated on the last vertebrae (Cv8). None of the anterior portions of the osteoderms are preserved, being lost or covered by the ones in front. The dorsal ridge extends almost to all the dorsal area of the osteoderm, being slightly high anteriorly and decreasing posteriorly up until the posterior border. They share overall similarities to the ones in *Prestosuchus* (Azevedo, 1991; Mastrantonio, 2010), *Ticinosuchus* (Krebs, 1965; Lautenschlager & Desojo, 2011), *Rauisuchus* (Lautenschlager & Rauhut, 2014), *Teratosaurus* (Brusatte et al., 2009) and *Yarasuchus* (Sen, 2005).

**Figure 17:**
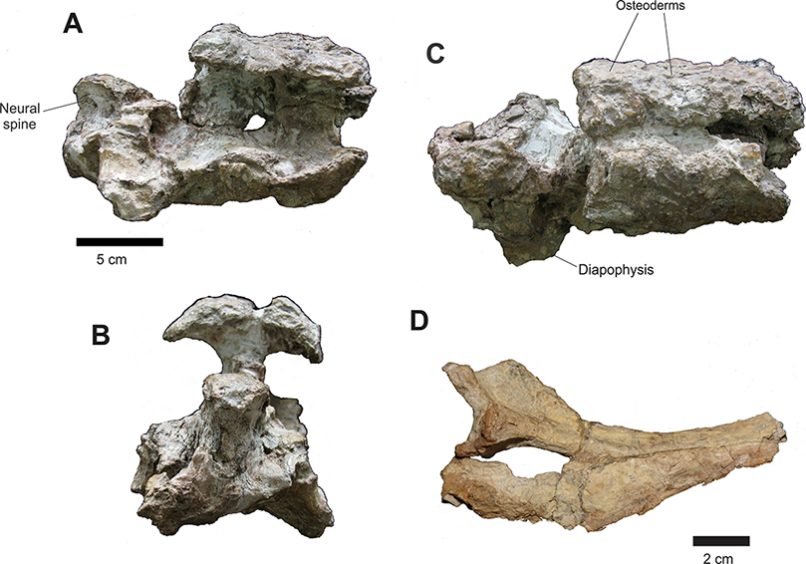
Osteology of *Prestosuchus chiniquensis* specimen CPEZ-239b, disarticulated vertebrae and ribs. (A) lateral, (B) anterior and (C) dorsal views; (D) incomplete cervical ribs in lateral view.

Two incomplete cervical ribs are present in this assembly (Fig. 17). In lateral view, their shape is elongated, slender with a slight posterior curvature. The proximal portion of the second rib displays an expansion that forms the capitulum and tuberculum where a small blade extends posteriorly and follows the ventroposterior border of the rib, tapering gradually in until it ends near the middle portion of the rib.

### Appendicular skeleton

#### Girdles

**Figure 18:**
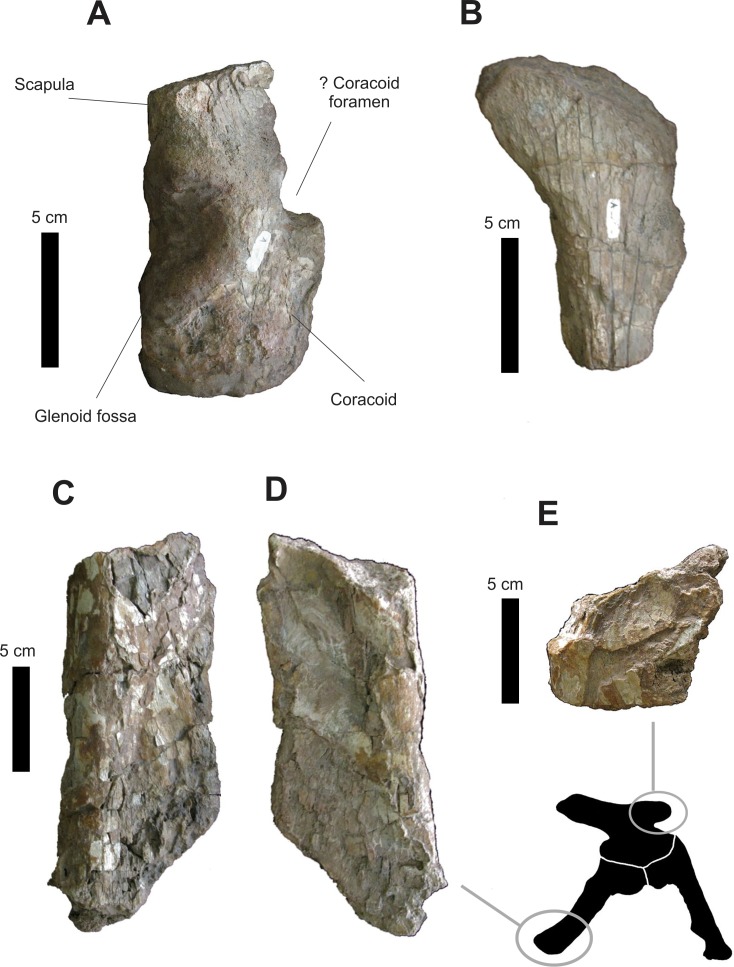
Osteology of *Prestosuchus chiniquensis* specimen CPEZ-239b, preserved fragments of the shoulder and pelvic girdles. (A–B) fragments of the shoulder and (C–E) of the pelvic girdle.

Of the shoulder girdle, an articulated portion of the right scapula and coracoid is preserved, which corresponds to the area around the glenoid fossa. Additionally, there is a fragment of the scapular blade, but it is not possible to discern from which side (Fig. 18).

In lateral view, the fragment of the scapula+coracoid has a sub-rectangular shaped anterior portion with a dorsal margin that continuous dorsally to a semi-circular fracture that is on the central third of its body. After this fracture, the dorsal margin follows posteriorly at a more ventral level than its parallel margin. The ventral edge of the coracoid continues straight anteroposteriorly until the thick edge of the glenoid fossa. This, in turn, is concave, semi-lunar shaped and occupies much of the ventral portion of the bone. The preserved portion of the posterior border of the coracoid is relatively dorsoventrally straight.

In medial view, the coracoid presents no remarkable feature, with only one lateral thickening in its latter portion. The ventral edge of the glenoid fossa is ventrolaterally orientated and directed posteriorly, with its entire anterior margin next to a thickening located above the glenoid fossa. Posterior to the glenoid cavity is a fracture with rounded edge which corresponds to the opening of the coracoid foramen. The portion where the anterior notch would be is not preserved (Desojo & Rauhut, 2009).

A piece of a left scapula, in lateral view (Fig. 18B), is expanded anteromedially with all its edges damaged. The anterior border is sinuous and continues dorsally to a posterodorsally convex area that forms a slightly rounded area, occupying much of its dorsal portion. After this, the margin follows ventrally to an area with a fracture slightly rounded that is located on its ventral portion. In anterior view, the scapula fragment is a lateromedially compressed blade, which has a medially orientated curvature.

The only elements present in the pelvic girdle of CPEZ-239b are an incomplete ventral portion of a left ischium and a fragment of the anterior process of a right ilium (Figs. 17C, 17D and 17E). In dorsal and ventral views, the fragment of the ischium is roughly sub-rectangular shaped and slightly elongated. Its lateral edge is rounded where it expands medially.

The fragment of the anterior process of the ilium, in lateral view, shows a sub-rectangular shape, longer than tall, with its dorsal edge irregular and slightly rounded. In anterior and posterior views, the ventral portion of this fragment expands medially to form a flat surface that occupies the entire area of the dorsal bone.

#### Propodial elements

**Figure 19:**
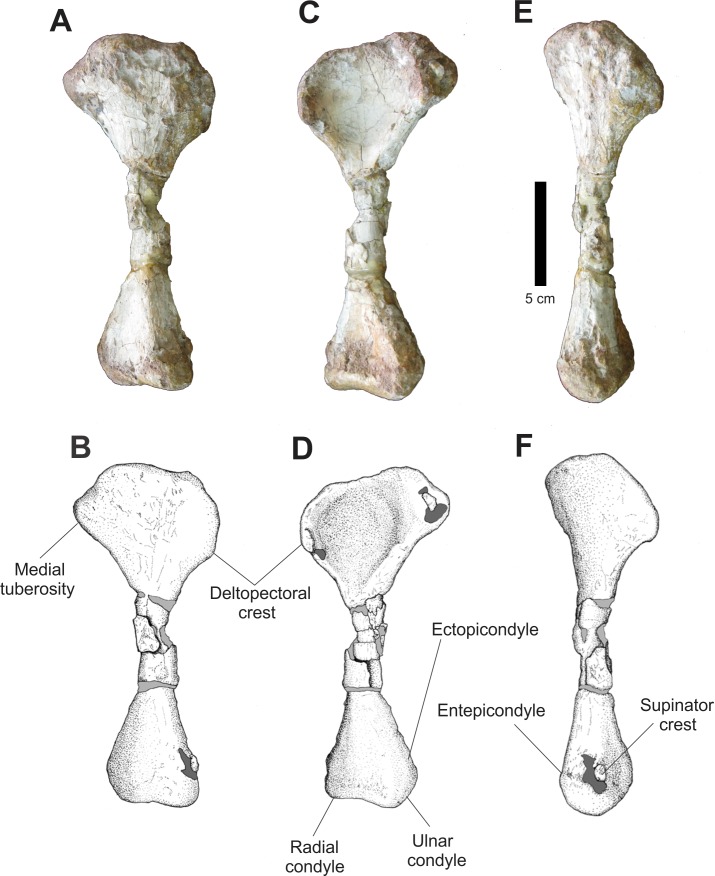
Osteology of *Prestosuchus chiniquensis* specimen CPEZ-239b, right humerus. (A–B) lateral, (C–D) medial and (E–F) and anterior views.

Only a complete right humerus is preserved in the assemblage (Fig. 19). In anterior view, the anterior border of the proximal humerus is irregular, with a prominent deltopectoral crest. The proximal margin is convex and irregular, with a large and thick posteromedially located area and a prominent medial tuberosity. Between these two structures there is a depression across its anterior face. Distally, the humerus tapers lateromedially in a very fragmented diaphysis and expands again in its distal portion, forming a slightly convex distal margin where there is radial and ulnar condyle.

In posterior view, the torsion between the proximal and distal epiphysis is clearer. All the proximal border of the proximal epiphysis is irregular and slightly compressed transversally. The proximal epiphysis, in lateral view, possesses a lateral margin with an attenuated anterior curvature and a distinct deltopectoral crest. In its distal end, there is a small supinator crest, located near to the radial condyle.

Damage on the surface of the humerus by chemical preparation makes it impossible to clearly ascertain if the condition of the epiphysis would be un-ossified, a feature that has been used to establish ontogenetic variation within *Prestosuchus chiniquensis* (Mastrantonio, 2010).

The humerus of *Prestosuchus chiniquensis* (Mastrantonio, 2010) and *Ticinosuchus ferox* (Krebs, 1965) are the closest matching the one of CPEZ-239b. However, we must consider that most of the humeri described are morphologically similar and diagenetic alterations are rarely discussed in most works, which complicates precise identification.

**Figure 20:**
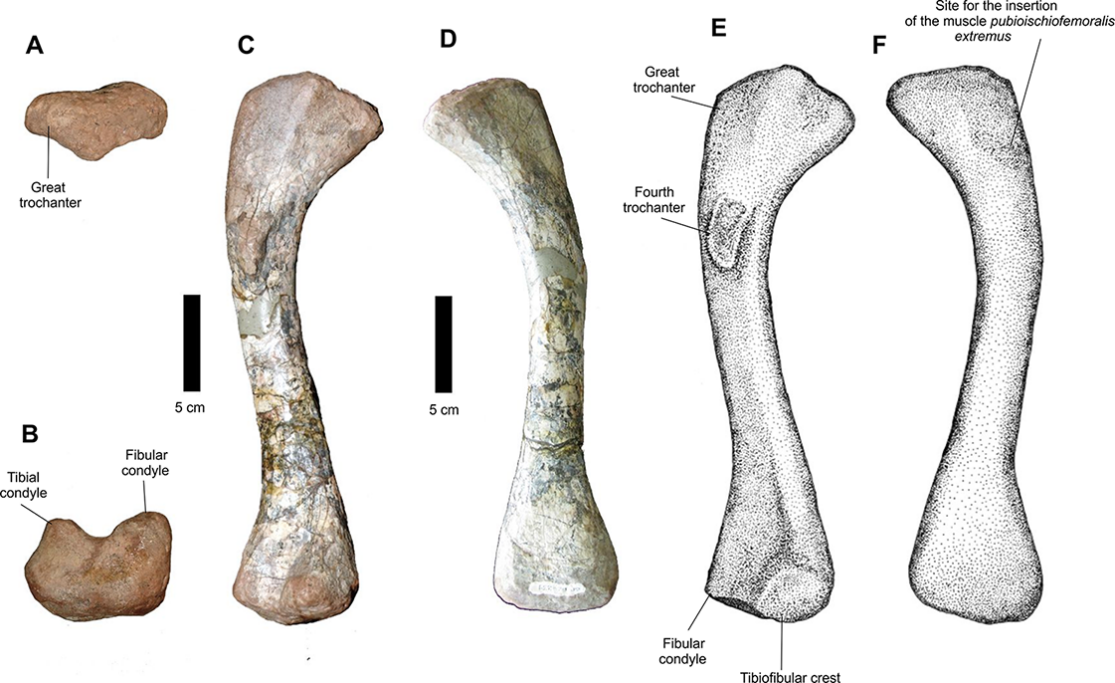
Osteology of *Prestosuchus chiniquensis* specimen CPEZ-239b, left femur. (A) proximal, (B) distal, (C–E) medial and (D–F) and lateral views.

Of the posterior propodium, a well preserved left femur (Fig. 20) and a distal portion of a right one are present in CPEZ-239b. The more complete one is an elongated bone, with a robust proximal epiphysis and a distal anteroposteriorlly compressed one. The trochanter is not well marked, such as the ones present in other specimens of *Prestosuchus chiniquensis* (Huene, 1935–42; Mastrantonio, 2010). The torsion of the proximal area turns medially. This area displays a well-defined medial incline, while the greater trochanter has a smooth margin. In dorsal view, the articular surface is anteroposterior narrow and completely smooth. In posterior view, the proximal region displays a short projection that would correspond to the central ridge (*sensu* Gower & Schoch, 2009). This ridge possesses a rough surface, which extends dorsally to the articular area and has, in dorsal view, a triangular aspect with a rounded apex. Located ventrally to this elevation there is the fourth trochanter, which is projected laterally from the body of the femur, is a dorsoventrally elongated structure and is positioned obliquely to the axis of the femur. Its structure, in medial view, presents a broad proximal margin, which tapers slightly distally.

A rough surface on the anterolateral area of the proximal end, close to the anterior margin of the greater trochanter, continues across a quarter of the total length of the femur. This area corresponds to the insertion site for the *pubioischiofemoralis externus* muscle. The distal articular region is divided in to a fibular condyle, in lateral position, and a medial tibial condyle, both separated by a shallow longitudinal intercondylar groove.

The preserved fragment of a right femur is a distal epiphysis, anteroposteriorly compressed and damaged. Although it does not display any features, its overall form makes it possible to identify it as a right femur, based on the based on its dorsoventral expansion when compared to the most complete femur of the assemblage.

Comparatively, the best preserved femur of CPEZ-239b is similar to the ones of *Prestosuchus chiniquensis*, especially the rugose area on its anterolateral surface However, it is more slender than the ones described on the aforementioned work and the sigmoidal torsion is not as pronounced, but this can vary even in the same specimen due to diagenetic factors (Mastrantonio, 2010).

#### Epipodial elements

**Figure 21:**
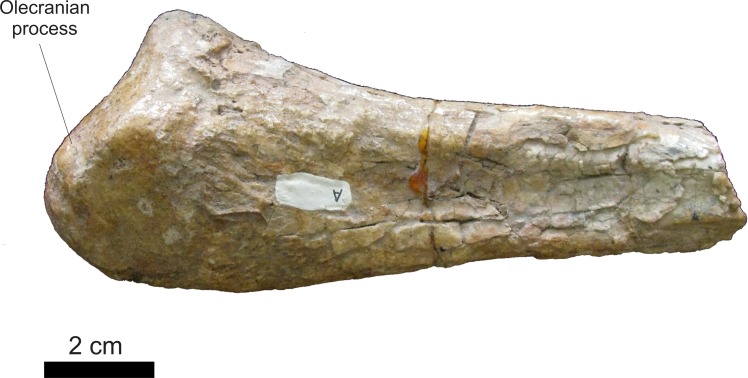
Osteology of *Prestosuchus chiniquensis* specimen CPEZ-239b, right ulna. Proximal portion in lateral view.

Among the elements of the forelimb, only a fragment of a proximal right ulna is preserved (Fig. 21). In medial view, the distal portion is elongated, with the two margins running parallel up to the proximal area, where the anterior margin expands anteroposteriorly to form the olecranon process. In lateral view, the olecran is concave and low, located proximally to the dorsal surface (*sensu* Gower, 2000), similar to the one in *Ticinosuchus ferox* (Krebs, 1965), but differing from taller ones, such as those of *Postosuchus kirkpatricki* (Weinbaum, 2013), *Postosuchus alisonae* (Peyer et al., 2008), *Fasolasuchus tenax* (Bonaparte, 1981) and *Prestosuchus chiniquensis* (Mastrantonio, 2010).

**Figure 22:**
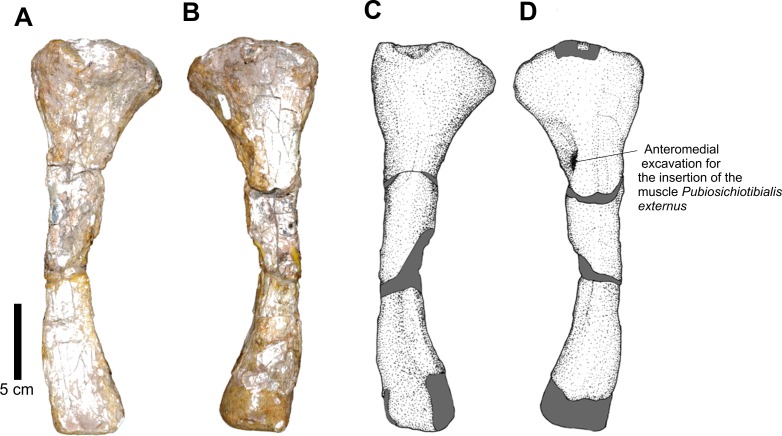
Osteology of *Prestosuchus chiniquensis* specimen CPEZ-239b, left tibia. (A–C) anterior and (B–D) posterior views.

A left tibia is also present in this material (Fig. 22). In lateral view, its proximal end is expanded lateromedially, tapering near the diaphysis and maintaining the diameter similar to its distal end. Its most distal portion is incomplete. In cross section, the shape of the diaphysis is oval, whereas in lateral view, there is a lateromedially pronounced curvature.

At the distal end of the proximal area, shortly after its expanded proximal portion, there is an anteromedial groove, which would correspond to the insertion site of the *puboischiotibialis externus* muscle.

#### Autopodial elements

**Figure 23:**
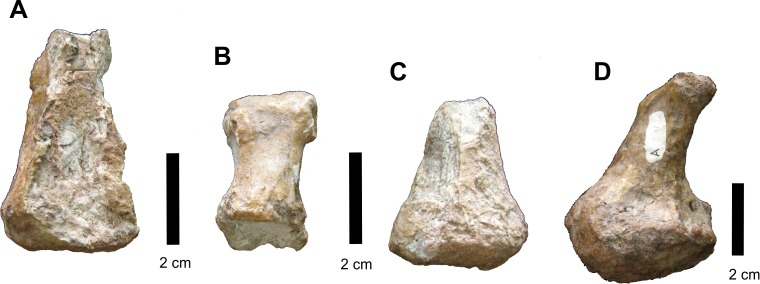
Osteology of *Prestosuchus chiniquensis* specimen CPEZ-239b, autopodial elements. (A–D) different phalangeal elements of CPEZ-239b, including (D) the “hooked” 5^th^ metatarsal.

Some incomplete phalanges are preserved attached to the side of the right disarticulated mandibular ramus (Fig. 23) but were separated during preparation. These are small, slightly cuboid bones that range from 5 to 3 cm in length and it is unclear from which autopodium these phalanges belong. A single right fifth metatarsal is preserved, displaying the characteristic hooked shape.

### Phylogenetic analysis

The phylogenetic analysis was made using the data matrix of Nesbitt (2011) altered to include CPEZ-239b as an Operational Taxonomic Unit (OTU) and following the same parameters (a heuristic search of 1000 replicates of Wagner trees with TBR branch swapping, holding 10 trees per replicates). The OTUs *Prestosuchus chiniquensis*, UFRGS-PV-0152-T and UFRGS-PV-0156-T were removed from the analysis and only “Combined *Prestosuchus*” was used since all 4 OTUs formed a polytomy both in Nesbitt’s (2011) and in our own initial tests. Nodal support was tested in TNT by using Bremer support and bootstrap resampling parameters.

**Figure 24:**
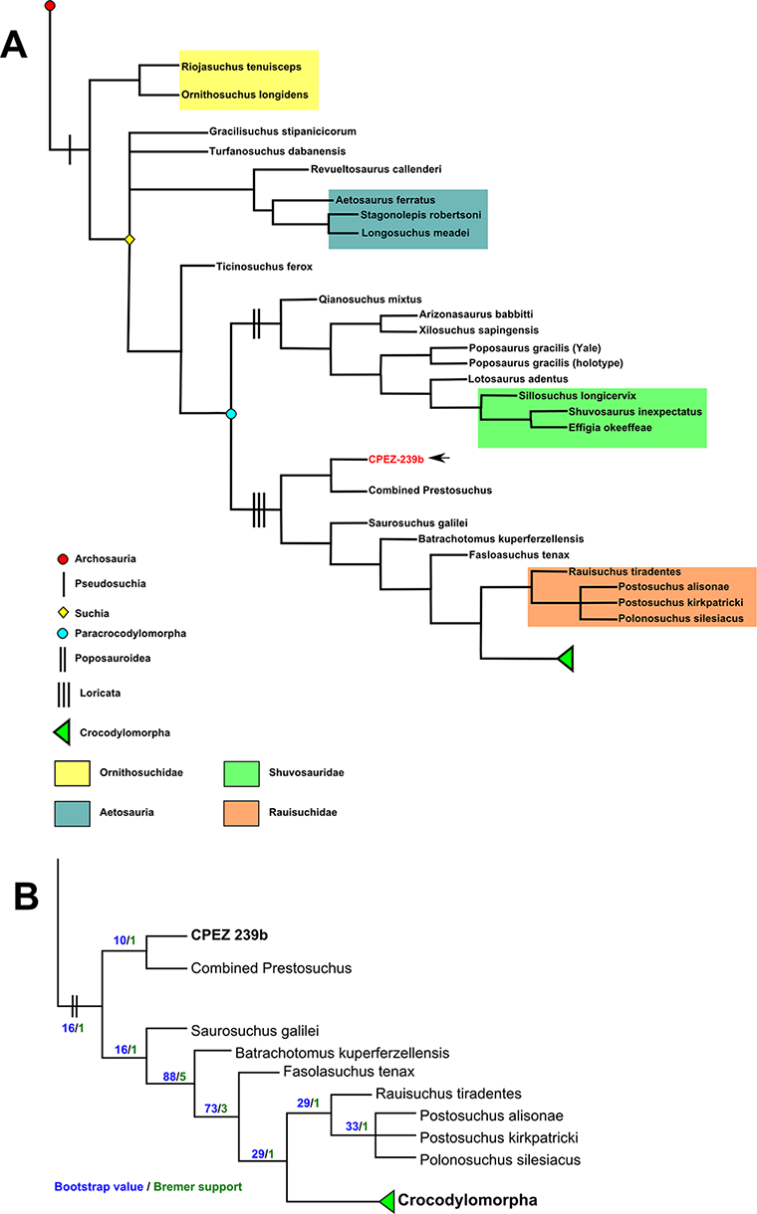
Phylogenetic analysis results. (A) Portion of the consensus trees of the analysis indicating the CPEZ-239b (indicated by the arrow) as the sister taxon of “Combined *Prestosuchus*”; (B) Bootstrap and Bremer support values tree.

The analysis resulted in 360 equally Most Parsimonious Trees (MPT) of 1280 length steps (retention index [RI]: 0,778 and consistency index [CI]: 0,376). CPEZ-239b was recovered in all MPTs as the sister taxon to “Combined *Prestosuchus*” by only a single step and with good nodal support this result (Fig. 24).

Our initial tests found only one autapomorphy for CPEZ-239b character 31; Maxilla, anterolateral surface, large anteriorly opening foramen: (0) present; (1) absent (Nesbitt, 2011:66) with “Combined *Prestosuchus*” being (0) and CPEZ-239b (1). However, this result was problematic. No such structure is present in the three specimens that form “Combined *Prestosuchus*.” The lectotype and paralectotype do not have preserved maxilla, UFRGS-PV-0156-T and UFRGS-PV-0152-T both do, but do not possess any feature similar to the one described for this character state. As such, we altered this state in “Combined Prestosuchus” from 0 to 1, thus turning character 31 to a sinapomorphy of Combined Prestosuchus+CPEZ-239b and not finding any autopomorphies for the latter, thus indicating that both OTUs would belong to the same taxon.

## Results

Both the comparative morphological and the quantitative phylogenetic analyses indicate that CPEZ-239b can be attributed to *Prestosuchus chiniquensis*. Although it presents features that differ from other specimens described for this taxon,the resulting strong support for the taxonomic attribution would indicate that these are likely due to some form of intraspecific variation. The first and most obvious factor would be the smaller size of CPEZ-239b in comparison to all the specimens that compose the “Combined *Prestosuchus*” of Nesbitt (2011) and UFRGS-PV-0629-T, which would be too great to be considered as sexual dimorphism. The most likely possibility would by ontogenetic variation, but the problem in establishing this in *P. chiniquensis*, or in any other ‘rauisuchian’ and Triassic pseudosuchian taxa, must be addressed.

## Discussion

As presented by Irmis (2007), size alone is problematic when used to determine ontogenetic variation within archosaurs. Very few taxa are represented by a large number of mostly complete specimens to clearly test for intraspecific variation (*e.g.* Brochu, 1992; Irmis, 2007; Schoch, 2007; Nesbitt et al., 2013). This is not restricted to Triassic forms, being intrinsic to the study of fossil organisms, but the chance to clearly view this within a fossil vertebrate group is very rare (*e.g.* Brinkman, 1988; Dodson, 1976; Colbert, 1992; Raath, 1992; Carr, 1999) and must be considered with uttermost caution.

In studies of ontogeny in the pseudosuchian lineage, living crocodilians are logically considered as proxies to the extinct forms (Brochu, 1992; Brochu, 1996; Rieppel, 1993; Irmis, 2007; Ricqlès, Padian & Horner, 2003; Ricqlès et al., 2008), although this also is not without its problems (see Irmis, 2007, for a more detailed discussion on this topic). In the case of ‘rauisuchuans,’ there are descriptions of juvenile forms in the literature based mainly on morphological features. Desojo & Arcucci (2009) described a partial skull roof and palate (PULR 057) that they attributed to a juvenile form of *Luperosuchus fractus*, based on its size (one third of the type specimen PULR 04), the loose condition of its sutures, comparatively reduced ornamentation on the surface of the bones and a more pronounced excavation between the premaxillae and maxillae. Chatterjee & Majumdar (1987) in their description of *Tikisuchus romeri*, found that its skull would be proportionally longer than the pre-sacral length of its axial skeleton and this, along with clearly defined sutures among some elements of the skull and the disjointed neural arches, would indicate poor ossification, which the authors concluded would be evidence of an immature individual. Gower & Schoch (2009) described the postcranial skeleton of *Batrachotomus kupferzellensis* based on four small and one large individuals. These authors did not discuss the presence of sexual dimorphism and individual variation among described specimens, but indicated that the smaller forms displayed minor differences in the pelvic girdle and locomotor appendages, in comparison to the larger specimen (specifically longer and more robust pubic boots in the larger specimen, ilia with less developed rugosities on the iliac blade and slender limb bones with less developed trochanters in the smaller ones).

In the description of *Arizonasaurus babbitti* (Nesbitt, 2005), other incomplete specimens were attributed to the species, but the author did not discuss any case of variation, possibly due to the incomplete state of the material. França, Ferigolo & Langer (2011) and França, Langer & Ferigolo (2013) described *Decuriasuchus quartacolonia* based on 10 incomplete individuals, which shared approximate dimensions and were identified by the authors as adult forms due to the closed neurocranial and neurocentral sutures (França, Ferigolo & Langer, 2011:390–391). Outside of ‘rauisuchians,’ specific work on ontogeny was made for phytosaurs, aetosaurs and proterosuchid archosauriforms (Martz, 2002; Irmis, 2007; Lucas, Heckert & Rinehart, 2007; Parker, Stocker & Irmis, 2008; Ezcurra & Butler, 2015). Alternatively to macroscopic features, histological studies have been done, being used to establish age estimations in crocodylomorphs (Erickson & Brochu, 1999; Hill & Lucas, 2006; Ikejiri, 2012), aetosaurs (Parker, Stocker & Irmis, 2008; Cerda & Desojo, 2011; Taborda, Cerda & Desojo, 2013) and ‘rauisuchians’ (Ricqlès et al., 2008; Taborda, Cerda & Desojo, 2013).

In the case of *Prestosuchus chiniquensis*, ontogeny is mainly inferred by comparative means. Azevedo (1991) considered the specimen UFRGS-PV-0156-T to be of an adult form due to its size (skull length about 1 m long), while Mastrantonio (2010) proposed that specimen UFRGS-PV-0629-T would be of a sub-adult, based on its comparatively smaller size relative to the one described by Barberena (1978) and Azevedo (1991). Additionally, these authors also consider that the condition of the articulation of the skull elements as ontogenetic: UFRGS-PV-0156-T has tightly fused elements while the disarticulated condition of the ones of UFRGS-PV-0629-T would indicate that its sutures were not completely closed, thus indicative of an immature individual. In CPEZ-239b, the disarticulated condition of the cranial elements are similar to those of UFRGS-PV-0629-T (Mastrantonio, 2010), and the ones of the dermal roof display clear sutures, such as the one of the frontal-parietal, that would indicate a weak ossification. This is further supported by the distorted condition of the neurocranium and the disarticulated mandibular elements.

**Figure 25:**
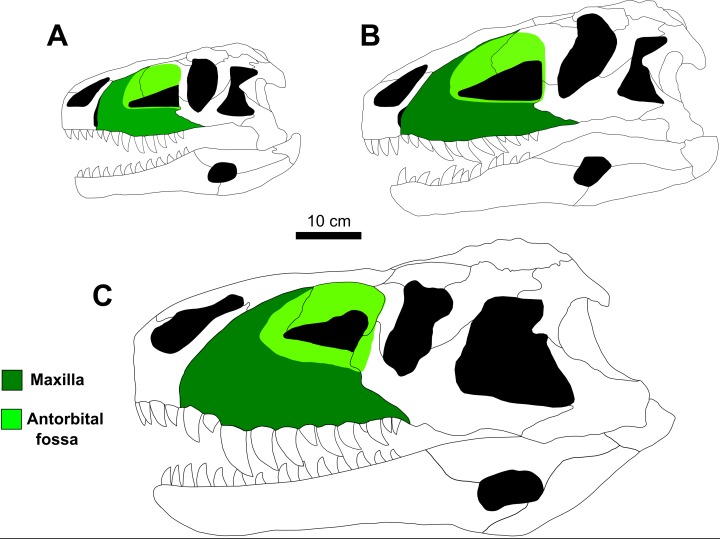
Skull comparasion diagram of the inferred ontogenetic sequence in *Prestosuchus chiniquensis*. (A) reconstruction of the skull of CPEZ-239b; (B) the skull of UFRGS-PV-0629-T (redrawn based on Liparini (2008)) and (C) UFRGS-PV-0156-T (redrawn based on Barberena (1978) but modified to correct diagenetic distortion and with an open jaw, reconstructed based on the mandible of UFRGS-PV-0629-T). The dimensions of the maxillae and of the anterorbital fossa are highlighted to indicate the apparent changes that occured in these elements as the animal aged and the impact this would have on the size of the sub-narial fenestra.

The absence of a subnarial fenestra in *P. chiniquensis* has long been inferred based on its lack on the complete skull of UFRGS-PV-0156-T, but the possibility of one being present but closing as the animal aged was considered by some workers (*e.g.* Alcober, 2000). However, clear evidence of its existence was only detected in UFRGS-PV-0629-T (Mastrantonio, 2010). As we present here, a similar structure does exist in CPEZ-239b, even with the anterior portion of the rostrum distorted. However, its exact dimensions must be considered with caution, since this site would represent some degree of kinetic articulation and thus be more susceptible to suffering distortion during fossilization (Gower, 2000; Liparini, 2008; Mastrantonio, 2010), but when comparing the size of the anterior border in CPEZ-239b relative to the overall dimensions of the premaxillae and maxillae in UFRGS-PV-0156-T and UFRGS-PV-0629-T, it would appear to be comparatively larger than the one in the latter. Additionally, it appears that what would regulate the dimensions of the opening would primarily be the growth of the maxilla. This is observed when comparing the area of the antorbital fossa of the three aforementioned specimens relative to their dimensions in the maxilla (Fig. 25). CPEZ-239-b and UFRGS-PV-0629-T are relatively similar, but the one in UFRGS-PV-0156-T is greatly expanded and the area of the fossa is comparatively reduced. This is of course a tentative inference based only on three specimens and remains to be quantativly tested, but require a larger sample size of specimens which is impossible at this time.

The cervical vertebrae of CPEZ-239b do not display any sutures, so are fully fused. Alternately, the three incomplete vertebrae that lack the centra possess sutures that are well clearly marked. The absence of centra would indicate that the suture between them and the neural arches was not a strong one, therefore these 3 other vertebrae would belong to another portion of the axial skeleton other than the cervical sequence, although the exact position is impossible to establish.

The epiphysis of the appendicular bones of the propodium of the smaller specimen are not ossified (Mastrantonio, 2010), which would be additional evidence of a younger ontogenetic state. Furthermore, these authors also state that the specimens described by Huene (1935–42) displayed appendicular elements in similar condition, which would indicate that both would belong to the same ontogenetic stage. Since the size of all the bones present in CPEZ-239b would be smaller than the ones of the above-mentioned specimens (Huene, 1935–42; Huene, 1942; Azevedo, 1991; Mastrantonio, 2010), this would further corroborate our size/ontogenetic stage correlation hypothesis.

Further evidence of a young ontogenetic stage for CPEZ-239b comes from Cerda et al. (2013) histological study, where an osteoderm of this assemblage was used along with other South American ‘rauischians.’ The estimated age for CPEZ-239b, assuming that the preserved growth marks were annually deposited would be 6 years, while UFRGS-PV-0629-T would be 8 years old and the *Saurosuchus* specimen PVSJ 32 would have 16 years. The authors were unable to estimate the age of UFRGS-PV-0156-T due to extensive secondary remodeling of the sampled osteoderm core (Cerda et al., 2013).

## Conclusions

Even with the incomplete condition of CPEZ-239b, both the comparative morphological study and the phylogenetic analysis indicate that it can be assigned to *Prestosuchus chiniquensis*. The differences between this material and other specimens described for this taxon are minor and did not cause any significant changes in the resulting taxonomic determination. Therefore, this would indicate that these variations would be intraspecific in nature. Based on the corroborative evidence presented and discussed here, we conclude that CPEZ-239b would be composed of juvenile aged individuals and this along with the presence of a subnarial opening in this material corroborates the hypothesis that such a structure is present in *P. chiniquensis* but it would close or greatly reduce as the animal aged. However, this conclusion is not ideal, since a quantative analysis to ascertain the different steps that occurred during ontogeny to better determine it is needed to clearly verify our conclusions, but unfortunately this is beyond reach at the present time due to the very low number of described specimens and the previously discussed need for the taxonomic revision of this taxon.

## Supplemental Information

10.7717/peerj.1622/supp-1 Supplemental Information 1 Data Matrix.

10.7717/peerj.1622/supp-2 Supplemental Information 2 Measurement parameters 1. Measurement parameters 1.

10.7717/peerj.1622/supp-3 Supplemental Information 3 Measurement parameters 2. Measurement parameters 2.

10.7717/peerj.1622/supp-4 Supplemental Information 4 Measurement Table. Measurement Table.
