# Supplementary material for: New insights on Prestosuchus chiniquensis Huene, 1942 (Pseudosuchia, Loricata) based on new specimens from the “Tree Sanga” Outcrop, Chiniquá Region, Rio Grande do Sul, Brazil
Source: PeerJ. 2016 Feb 1;4:e1622. doi: 10.7717/peerj.1622 (PMC4741083; doi:10.7717/peerj.1622)
Supplement: Supplemental Information 1 [file peerj-04-1622-s001.docx]

Mesosuchus_browni

?1000-0000000-1000000-0000-00-100000-00000000-000000000001000000000--0-000000000000000000?0000010000000000000000000???000?00010-0?0??000-000?000000--?00000000000-?000001--0-0010??00?000100000?000000000000000000000000000010-000000-???0??0000?0000000?00????00?0000?00--0-00000000000000--00000000000000---00000000000-0--000000000000000000000--10001000??0??00000-0000000--0000000000-00000000000?0000?00000----------0

Prolacerta_broomi

0000020000?00-0000000-0000000-100000-000000000000000000000000000000--0-000000000000000000?0000000000000000000000000000000?00000-?00??000-0000000010--100000?00000-000000000000000000101100000000000010000000000000000000000010-000000000000000000000000000000000000000?00--0-00000000000000--00000000000000---00000000000-0--000000000000000000000--100010000????-0000-0000000--0000000000-0000000000000000?10000----------0

Archosaurus_rossicus ????03010???????????????????????????????????????????????????????????????????????????????????????????????????????????????????????????????????????????????????????????????????????????????????????????????????????????????????????????????????????????????????????????????????????????????????????????????????????????????????????????????????????????????????????????????????????????????????????????????????????????????????

Proterosuchus_fergusi

000003010000000000000-0000200010000000000000000000000000000000100000?130000000000000000100010000000000000000000000000?00000001000000?00101001100000--100?00000000000000100000000000-00000000000000001000000000000000000?000010-000000??0?0?00000?000000??000?0?0??0000?00--0-00000000000000--00000000000000---00000000000-0--000000000000000000000?0100000000000000000-0000000--0000000000-0000000000000000?10000----------0

Erythrosuchus_africanus 0100020000020000000000000000000000000000?0000000000000000000001000001101000000000000000100011100100000000000000000000000010000000000000111000100100--000000000000001000100000111000-00?00000000?00010000001000000000???0100100-0000001?0001001-00??0?00??01??0????0?00?00--1010000000000000--000000000000000100000000000010--0000000000000000000000010001100000?000000-000?0?0001000000000-010000?000000101?000?0----------?

Vancleavea_campi

1100020000100-100000000000-00-000000---0-00100000-00000?00000010-000-10000000100000????????1???????????0?00000???00??0???????10??????00--00100--110--10?0000?0?00-?10011000001??1???00?001?00?000?0?0000???000000000???010010?????0??00001000000000000???00??0?0??010??00--0-00000000?000????00???0000000-0010000000000000???100?00000000000000000??1000110??????-0000-0001000001000000000-01000000?0?00????00??1?1?100011?2

Chanaresuchus_bonapartei 10000200000001000000000000000000000000000001001100000010000000100000010000100000000000010?0?11011000000000000000000000000??0??0???00000111010000110--0000000000000010001000001011000000000000000000000000010000000?0???0100100-000000000010000000??????????????????????00--1000000000000000--0000000000000001000100000000110010000000000000000000100101011000????00000-00000100010000000?0-010000001000011000020100-0-0001-?

Tropidosuchus_romeri 10??0?000000000000000?0000?0000?0000001??001001100000010000000100000?100001000000000?001??????011?00?0?0?00000??0?0?????0?????0??????0011101?000110--00??00??0??00?1?001000?010110001000000000000??0000000100000000?0??0100100-0000000000100???????????????????????????00--1-00000000000000--000000?000000001000100000000010010000?00000000000000100101011000???000000-0001010001000000010-0100000010000110?0020100-0--000-?

Euparkeria_capensis 0100000000?000000000000000100010000000000000000100000000000000100000[0 1]100002000000000000100011101100000000000000000000000001001000000000111000000100--0000000001000010001000001010000000000000010000010000010000000000010100000-000000000010000000000000??010?0?00?0100?00--1000000000000000--000000000000000100010000000001001000000000000000000000000?001000000000000-1000010001000000110-0100000000000100?0000110100000000

Parasuchus_hislopi --00030001000110001000000020000000000000?000010100000?0000000010000001000000100000000001??0111121100000000000000000????000?00?????0??0011111?000110--100?00?00?100?1?011000001111?0000?00000001000011000001000000??00010001000-001000000000000000?00000??00????00?0?00?00--10?000?000000000--00?0000000000001000?00000000010010?00?0?00000-?0000001000100100??0??00000-?000010?110?0010111-11000000000001000?0011100?00000?0

Smilosuchus_gregorii --00030001000110001000000020000000000000000001010000000000000010000001000000100000000001??011?1211000001?0000000000???000??0?10???0??00111210000110--100-0000011000100110000-1111???00?0000000100001?0000010000001000010100000-001000000000000000??????????????????????00--1000000000000000--000000000000000100000000000001001000000000000-00000001100100???????000000-1000010011010010111-1100001????00????00?21100??0000?0

Pseudopalatus_pristinus --0003000100011000100000002000000000000000000101000000000000001000000101000010000000000?0?011112110000010000000000000000001001000000000111210000110--100?000001100?100110000-111100000000000001000011000001000000??00010100000-00100000000000000???000???00000?0???100?00--1000000000000000--0000000000000001000000000000010010000000000000000000011001001000001000000-1000010011?10010111-1100001000000100?0002110010000010

Gracilisuchus_stipanicicorum 0100000000000000000000000010000?0000100000000001000000000000001001100111002001000000?001????1?2211010010??0000?????????????0??0??????00121000000110--00??00000?0000100010000011110000?0000000-1000?1?0000010000000?0????1??????????????????????????????????????????????00--1000000000100010--00000010000000?1000?0000000001001?000?00000???000001000???00100000??00000-1000011011011010221-1100000000000100?010011100000011?

Turfanosuchus_dabanensis 0003020000000000000000000020000?00001000?0000001000001000000?01000001100001000000000??0?0???110211010000000?0000020????001100?00?00??0012100?000110--00??000?0?000?10001000001011?????????00001?000?1??0001000000?????????1??????????00000001??????????????????????????00--1000000000000??0--0??00????????011000100000000010010000?0000001000000?010001??????????????0?????????1101101?221-1????????????????????1110??00?1??

Ornithosuchus_longidens ??00000100001000000000000000?001100?0000?000000100000?0000000110000001000000000000001001000???????????????0000????????????????0??????00121000200110--00?000000?1000?0001000001111??????0??0001100???10100?10?0?0?1?000101001010010000000??00???0?0?0100???0??0???????0?00--1000000000100010--00?0001000000?11000?00100000110010?00?0?0?0?1000000??1????0???????????0?????????1?21010010120-??000?????????????1?2110000100010

Riojasuchus_tenuisceps 00000001000010000000000000001101100000000000000100000000000001100000010000000000000010010?011?2211010000?000000??00?????0??0??0??????00121000200110--00?0000001100010001000001111000000000000111000?101000?010010???????100101011?00000001001100?0001000?00??0????0?0??00--10000000001000?0--0?000????000?011000100100000010010100?0000001-000001011001001000000100000-1000011021010010120-1100000000000100?010211000020001?

Revueltosaurus_callenderi 00030200000300110000001001201001000000000010000100000100000000100110112000200000000000010?01112211010000000000?00200??0002100??????0?0012100?00011???00000000010000100120000-111100000000100002000011010001000000000?0101001010010000000000011011000?0???000????0??????00--?000000000000000--0000000000000011000100000000010010000000000010000001000001001?00000100000-10000110110110102210110?000000?00??0?00??11001?211012

Stagonolepis_robertsoni 00000210000?00110000000111201001000??0?1000010010000010000001110011??1?001?0000001000001?101??221101000000?0001002?011000??00??01020?0012100001011???000?21101?000?101000010-1111???????0?000010001110100010000000??001?1011???0100000000000?????00010???01000000?0100?00--101000000000000???00?0000000000?11000?00000000010010?00?00000????0000??11001001????????00????????11???0?1??????-?10?00000?0??10?0000?11001121101?

Aetosaurus_ferratus 0000011000000011?0000?011020100?000?100??00010010?0001000??011110110112000200010?100000???????221?01?000?00000??????????0??0?1???????001210000101110000??21??1?000?101010010-1111??????????????????????0?0100000???000101?01010010000000000011011000?00??00000????0100?0[0 1]-?10?000000000000???00?00000000000110001000000000100100?0?00000?10?000010???0??0??????????????????????????1?1?2?1????????????????0????21?0011211012

Longosuchus_meadei ??001?????0?001?000000011020100?000000010000??01?0??01??0000?11???1?1????12000?00??0?00?0001?12211010000000000100200?10002100?00?02000012100001011???0?002110100000101000010-11110?00000??00001000??101000?000000??0???01011?10010000000000011011000100000000000?00100?00--1010000000000000--00?000?00000001100010000000001001000000000001000000101100?0010?00???00000-1000111011021010221-110000000000010??0002110011211012

Ticinosuchus_ferox ?????????????000?000000?0000010??????0?0???0?????????????????????????????????????????????????????????????????????????????????????????001210???0?????0?0??00?????00????01000001??1???0?0000?000?0?01110000??0000001000??010???100?00??0000?0011??0000100??000?0?00?0100?????10?0?0?000000?00--0??0?101100?1011??0?0??00??0?1001?????0???00?0?0000?000??100100?????000?0-1000011?11011010221-110000?000000100????2111000000100

Qianosuchus_mixtus 1000130000010100000100011010000100000000?000000100000000000000?000001100002000000000000??10???02??01?00????000?????????????0?????????0012100?000110--00??00??0??0001?001?00?01111?0?1?1000000000?010000?01??0010?10?001010010????0??00000?00???????????????????????????010?10?00200000000010001100100100010??000?00000000?1001?????000?00???0??0??00??00110????????000??00??1??11011010221-110000?00?000101?0002111?00000100

Xilousuchus_sapingensis ?0001200000?00000001000110000001??0??0??????????????????????????????????????????????????????1100110100000000?000000001?001100?00000?00?12100??????????000001101000??0001000001??1000?010010000000110???????????????????????????????????????????????????????????????????????????????????????????????????????????????????????????????????????????????????????????????????????????????????????????????????????????2????????????

Arizonasaurus_babbitti ???01???0?0??0000001000110000001?00??????000?00100?00000000000100000?100002000000000?0010???110011010000000000000000110001100?00000100012100?000?1?--?000001101000?1000100000111100010?0000000001110010?111000100???00??1001?10010000??????????????????????????????????01001010020000?00011100211010110001?10000100000000110011000100?0?????????????????????????????????????????????????????????????????????????0----------?

Poposaurus_gracilis_holotype ??????????????????????????????????????????????????????????????????????????????????????????????????????????????????????????????????????????????????????????????????????????????????????????????????1???0???1???1?0??????????????????????????????????????????????????????11111??0120000?????????????1011???-?10000?00000000?1001??????????????????????????????????????????????????????????????????????????????????????????????

Poposaurus_gracilis_yale ?????200??0?0???00??00????????????????????????????????????????????????????????????????????????????????????????????????????????????????????0???????????????????????????010?0001??1??????0???00-00101000011110111?00110??0?00??1001?000010000011000000100??0000000020100?1111110012?00010001110021101011000-01000010000000011001??0010000001-00000?0001010?1001001?00001-1000111011011011121-11000000?00?0101000[0 1]10-------0--0

Lotosaurus_adentus ?00004-0000200-0-1001-0011000010000000000000000000000001000000100000110001200000000?????0??111021111000000000000000????001100?00000??00121001000110--?0??0110110000102--------??1000000000?00000?11?0101?11?0010???0???0100000-0101000000000110?0?00100??0000000000100?11101?0002?01?????????01???2?10000-0???00?000000001100111?-?0000001-000?0??0000101????????00000-100001??11011010121-11000000000?0101?10010----------?

Sillosuchus_longicervix ????????????????????????????????????????????????????????????????????????????????????????????????????????????????????????????????????????????????????????????????????????????????1???10?0???1100000??0001111011110???????0????10000100??????1???????????????????????????11111?00121?101000111002?102?1?000??10??0??00?00?0?1001??0??0????????????????????????????????????????????????????????????????????????????0----------?

Effigia_okeeffeae 100014-000?200-0-1001-?111000000000000000011???0000000010020?01?0010????000000?01100?0010?01012211010010000?0?????1011000??00??0??0?00012100?000110--000?0100100011102--------111???00?01?0?1100?01?001111111011?0110??0000??10000110??0?101?1-00000100??00?00?0???????11111200021010?0001111021212010000-?30100??00000011???1110110000001-01000100010101100100110000?-1000011011011?1?121-1100000000110101?00?10----------0

Shuvosaurus_inexpectatus 100014-000?20?-?-1?0?-??????????0?0??000?0110001000000010020?0100010??00000000?01100?0010??101221101001000000100001011000??00100??0000012100?000110--?0000100100011??2--------111000000000011100????00?111111011??11???0??0??1000?1100000101110????????????????????????1111120002101010001111021212010000-030100100000001112-1110110000001-000001000101011001001100001-1000011011011010121-1100000000110101?00?10----------?

Prestosuchus_chiniquensis ?????????????????0?????????????1??????????????????????????????????????????????????????????????????????????????????????????????????????0??1????????????00?00????00??10001?00001??1????0?0?00000?00??????0??10?000????001?1?11010010001??00??????????????????????????????0?????10000000?00??100?1???10110?0?0110001000000001100100000000000?0000001011001001000000?0000?-1000011?11021010221-1100000000000101?10?2???????????0

UFRGS_0156-T 0100010000000000?0000?000010110?0?000000?00000010?11000?000?00100000?100000000?00000?00?0??1??2211010000?00100000?0?????0??0??0??????0?12100?100110--00?000010??000100010000011110000000000000?0001?100?????????????????????????????????????????????????????????????????????????????????????????????????????????????????????????????????????????????????????????????????????????????????????????????????????????1110?000?10?

UFRGS_152-T ??0???????0??0?0?0000?0000101101000?0?????0?00010?1?00000????0???0?0???0001000000000?0010??111221101000000010000020???0001100000?00??00121000?0?11???00000001010000??0?1000001111????????????????01?100000?0000001?????010?????0100??0000?00???????????????????????????00--10100000001000110001010101102010110001000000001100100?000000001000000?01100100????????????0?????????11021010221-1?000000000??????100?11?0?000?1??

Combined_Prestosuchus

0100010000000000?0000?000010111100000000?00000010?110000000?00100000?10000?000000000?0010??111221101000000010000020???0001100000?00??00121000100110--00000001010000100010000011110000000000000?0001?10000010000001??0010101101001000100?0?00???????????????????????????00-?1010000000100011000101010110201011000100000000110010000000000010000001011001001000000?00000?1000011?11021010221-1100000000000101?10021110?000?100

Saurosuchus_galilei 0100010000?00000000000?00010010100000000010100010011000?00000010000011??002000000000000100011122110100000100000002001??00?100100?00????12?0001001111100??0???????????00??00001111000000000000020001?1000?01000000??????????????????????????????????????????????????????010010100000001000?100010001011000??1?0001000000?0110010100?000000?000000101100?00100000??00001-100001??1102101?121-110?000000000101?10021110?000010?

Batrachotomus_kuperferzellensis ??0001001001000100000001100001010110100?01100001101?0000001000101000?10000200000000000011001112211010000010100000?00110001100100001110012100110111??000000011010000100010000011110000000000000200011100001100010010000?01011?1001?000000000011110??????????????????????01001010000000100011000100010010001010000100000000110011100000000010000001001001001?????????0?1???????1?110?1010221-1?0000000??00????10?21111?010?010

Fasolasuchus_tenax 01000100000101??0000010000100101?10?1?????????????????????????????????????????????????????????????????????????????????????????????????012?00??????????0000011?1???0??001000001??1?00000000000010001?100?????????????????????????????????????1110??????????????????????????????????????001????010?01???000?010000?00000010010011100?0000???????????0100110??01001110001-1000111111021011121-11???????????????????1??1?0??????

Rauisuchus_triradentes 010?01000?010????????????????????11?????0??????1101200000?????????00?1??003?01?????0?0011???????????????????0????????????????????????????100??0????????????1?010??0?0??10000?11110?100?0010000?0001?000??0??000?010?????101?????1?1?0??????????????????????????????????010010?0000000?00???????????????????????????????????????????????0????0000?00??01????????????00???0?0?1?1110??????????1???????????????????1111?011001?

Polonosuchus_silesiacus 01000100000100?000000100021011010110100?????10011?0200001??????0?100?111003?01000010???1111???????0????????????????????????????????????12?00?10???111?0012011?1??????001000001111001?0?000?000???????????????????1??????????????????????????????????????????????????????????????????????????????????????????????????????????????????????????????????????????????????????????????????????????????????????????????111??0?0????

Postosuchus_kirkpatricki 010001000001001000000100021011010110100?011010?1100200001010001011001111003001000010?0011??1112211010--011010010020011001??01??0???1?00121001101111110000201101000010001000001111??????????000?1?01?0000001000000??0???01001110?101000010000111?0?00100??00????0??0100?01001?10000000?????????1???1011000?0100001000000?0010011100?0000001000000100110110????????10001-1000111111021011121-1100000000110101?10?01111?01000??

Postosuchus_alisonae ??????????????????????????????????1?????????????1????????????????????????????????????????????1??????????1??1?0??????1???????1?0?????1?????????????111??????11?1???????010?0001??100100000?0000210011000???????????0?0???1???1101101000?100001111?00?100000000?000?0100????????????????????100??????????????????????????????????????????0?10?00001001101101001001110001-1000111111021011121-1100000000100101010101111?0100010

CM_73372 ????????????????????????????????????????????????????????????????????????????????????????????????????????????????????????????????????????????????????????????????????????????????1???0??0??0000?000?10000??100000?1??????1????????????0?????0?????0001000?00??0????0????010011?002?000100?11000??10101000-1010?00?000000100???1????00???0010000001001101101?01001110001-1000111111021011121-110000000011010-01010111100?000?0

Hesperosuchus_agilis ???????0?0????1?00?0000??0????02??0??????111???1?????0????1???????????????210110000????????????????1??-01?01?0??0111?????????????????0012??0??01??????00020??????????001000001??100000000000001?00?1??0??0??000?0??0???010011111101000011100111101?0?00??0?????????????010-111?001100?00??100???10????????01000010000001001001??1??00?000100000010??1011010010??1?0001???????1?1?0?10???21-110?000?00??0?0??002?1111?010?010

Dromicosuchus_grallator 0001020000110110000000000010010?0100001??11110011000001-00100010?1001111002?0110000?????????????????-??????100???????????????????????0012100?00111??00000?02101?00010001000001??1???????0??000000??10000?01000000?00201010011111101000011100011?0100100?10?????????????010-1110000100?00?????01???????????010000100000110010011100?0000001000000100010110?????????00?1???????1?11021011?21-1?1?000??????????????111100100010

Hesperosuchus_"agilis" 0001020000110110000000000010010201000011?111100110-0001-0010001001001111002?01100000?00111?1???????????0?1?100????1??1??1??????????????12100?00111110000?20210??00?100010000011110?00000??0000?00??1?0??????????????20101001?????????0?1?10011110100?00-100000000?0100???????????????1001?10001010????????0100001000000100???111??00???0???000?01?00101101001001110001-1000111111021011121-11000000001?0101?00??111100100010

Dibothrosuchus_elaphros ?0010200001?011000000000?0000002010000111111???11000001-0120101??1?01??????1011000000001??1111221101--10111100110111?1?11211010111???0?12100100111???00??002?0?0000100010000011110000000000000100001??0?????????????????10011111101000?1010011110110?00-100000?0??0100?00--1100021100??????????????????????????????????????????????????????????????????????????????????????????????????????????????????????????????1????????

Terrestrisuchus_gracilis ?????????????1100000000?00000002???000????111001110000?-??20?01??1001111000??1100000?00??0???????????-???11??0??0?1?????????0?0??????001210??00111????000002?0?00001?001000001111??00?000000000000?1000000100000010020111001111110100001010011110100100-100??0000?0100?010-1100021100100110--010001000002001000010100001001001010010000001000000100010?111001001110001-1000111111021011121-111000010?1001011011011??00100010

Sphenosuchus_acutus ?1010?00001?0110000000000010-?020100?011011110011000001-01201010010011111021011000000001111111221101---011110011011111111211010111111??12100100111???00002021010000?0001000001111?000???000000??00?1????????????????201010011111101000?1?100???????????????????????????????????????????????????????????????????????????????????????????00100000010???????????????????????????1???????????????100??????0???1?????1????0?????0

Litargosuchus_leptorhynchus 00010100001?011000000?00?000010?0000001??011???111??001??1010?1?0?001111102?01?0?0?0?0??????????1??????????110?????????????-?10??????00121001000111?000????2?0?0?0?10?0?000?01?11???00?00000000000?1000??????????0002?-11001?120101000010100?1?101?0???????????????????????11?????????????????????0?00????1??????????????????1??1??0?0?00?????????001???11?0????????????????1??1?0??0??121-1?1??000?????????012?1?1?000?001?

Kayentasuchus_walkeri ?10101000011011000000??000100102??0??01????????11110101-0?011?1????01??????101???????????011????????????1???1??????11???????0?0?????1?0121001?0?11???000101????1?0???00??000-1?????????????????????????????????????????????????????????????????????????????????????????0?????0??21?????????????????????????10?001?11000110100???1?????????????????????????????????????????????????????????????????????????????????1?????????

Orthosuchus_stormbergi 00020100001?01?0100000000010??020?00?011?01110011100101-?1011011?10??11100110111000000011?01-??21?10?--0?11110???11?????2??-??0??????0012100100011110000?202?0?000?1000?0010-11110?00000000000?000?10010?0100000???02??01001112010000001010011110110?10--00000000?0100?010?1100020100000-10--01--000000020010000000000010010010110?000000100000010???0?1110010???10001?1000?11111021011121-11??00???0??0????002?1110?021?01?

Alligator_mississippiensis 0-02020000000-10000-010000-0--020000---11011100110-010--01011011010--1110001011110000001---1-1221110-0-01100001101111111221-00011101?000-1011001111-000010000000000100000010-1111000000000000-110001001000100000010020101011012010000000110010111100100--000?000020100?-0----00021000000-00--01---0000002101000010-00011001001011000000001000000100010111[0 1]001001110001-1000111111011011111-110100000010011110020110011[0 1]?1011

Protosuchus_haughtoni 000201000011001000000?000010010?00000011?011100111--101-0101101?01001111102??111000000?1????-?221?10?0-0?11110????1?????2?????0??????0?1210010001111000??002101102?10?01000?011110?10?000??00000???1001?????????????????????1???????????????????????????1???????????????????????????????????????????????????????????????????????????????????????????????????????????????????????????????????????????????????????1110?021101?

Protosuchus_richardsoni 000201000011001000000??000100102000000111011100111-0101-0101101101001?11?0210111000000011001-?221110-0-01111101101111?1?2??-0001??1??001210010001111000??002101102?1000??00001111??10000000000?0?0?10010?0100000???0-011000111201000000?010011111110100???0?0000??0100?010?1100021100000?10--010-000000020010?001000000100100111100000000100?0001000101111?0100?010001-1000111111021011121-111000000010010110020111010211010

Eudimorphodon_ranzii 10-0?100000?00-000000-011000000?0?0?-0-?????0??????????????????00?0001000?0?0??0010??????????????????????????????????????????????????101?000?00?110--00?000000?00-0?001-0000-1011???1???00000000???100?00??????0?0100010??????1?1?0??0001100?0?00001-?-???0000101101-1?00--11???0?00?000000--00??00??000001????????0?00???-2-1??0??0?0??????0?????-??????11?00?????01???1???1???11-01----??111?00010?1?0101010000----------1

Dimorphodon_macronyx 10--1200000000--00000-011000000??????00?????0??10??0???0???????0000001??000??????0???????????????????????????????????????????????????1012100?200??0--00??00??0??0-?1?01-0000-1??1???1?1?????-000???1000??????????010???0??????101?0??0001100?????001-0-??00000??1101-1?00--11?000100?000?00--00??0???00000110000?000000000???1010020000000?00-000?00??1??111000?0-?01???101?1??-11-?1----??111000010?01?10??1?000-----?----?

Lagerpeton_chanarensis ???????????????????????????????????????????????????????????????????????????????????????????????????????????????????????????????????????????????????????????????????????????????????????????????????????0001000000??????????????????????????????????????????????????????00--1000000000000000--000000000000011110011000100?011010100?0010100?00000000010101111000?1-1010011010110?11-01----201111000000000000001200----------?

Dromomeron_gregorii ??????????????????????????????????????????????????????????????????????????????????????????????????????????????????????????????????????????????????????????????????????????????????????????????????????????????????????????????????????????????????????????????????????????????????????????????????????????1111001?01010010100101011011110000000000????????????????1??????????10??????-??????????????????????????????????????

Dromomeron_romeri ??????????????????????????????????????????????????????????????????????????????????????????????????????????????????????????????????????????????????????????????????????????????????????????????????????????????????????????????????????????????????????????????????????????????????????????????????????????1111001100010010-2-101?1101111000000000000101??????????-1010011010110-1?-01----2-1????????????????????0----------?

Marasuchus_lilloensis ????????????????????????????????????????????????????????????????????????????????????????????012211000000000??01?01000???0??00?00????0???????????????????????????????????????????10100?0000000000000?0000001000000000???01?0????0??1??000?1001???0??????????????????????00--1000001000000010--1100000000011110000100100101010010?00?000010000000001001010011100001-0110011000110-101000012001111000000000100?012?0----------?

Asilisaurus_kongwe ????????????????????????????????????????????????????????????????????????????0?00000??????????????????????????????????????????????????0????????????????000110000???0??1010?1?10??11?01010000000000010000000?00001000?????1?01?1001?100000?100??00???????????????????????02--1000010000?00??0--110?01001001??1000110111010111001010?1100010000010001??1110?????????-0110011000100-10100001?00111????????1?10?????10----------?

Eucoelophysis_baldwini ????????????????????????????????????????????????????????????????????????????????????????????????????????????????????????????????????????????????????????????????????????????????????????????????????????????????????????????????????????????????????????????????????????????????10?????????????????????????12001??11100001???1????1100010?100???????????????????????????????????????????????????????????????????????????????

Sacisaurus_agudoensis ?????????????010?0?0??000000000????????????????????????????????????????????????????????????????????????????????????????????????????????121????????????0?101??????????102001110??1???????????????????????????????????????1?01?????0100??????????????????????????????????02--??010?00001000?0--1???0?0???????10011101110000110010????100010010110001??????????????????????????????????????????????????????????????????????????

Lewisuchus_admixtus ?????????????01000?00?0?0000?00?????????????0001?0??000????????0001??1??000000?00001?011????012211001000000001??110????00?10??10??0??0?12??0?00?1?????01000??????????00100000?1111101?1?0000000000?0000?????????????????11010100101000?00??0????????????????????????????????????????????????????????????????????????????????????????????????????????????????????????????????????????????????????????????????????0----------?

Pseudolagosuchus_majori ???????????????????????????????????????????????????????????????????????????????????????????????????????????????????????????????????????????????????????????????????????????????????????????????????????0001000000??????????????????????????????????????????????????????02--??0?0100001000?0--?1010??????1?1100011?110010111?01010??000010000010001001?10?????????-0110011000100-10-00001?0011???????????????????0----------?

Lewisuchus/Pseudolagosuchus ?????????????01000?00?0?0000?00?????????????0001?0??000????????0001??1??000000?00001?011????012211001000000001??110????00?10??10??0??0?12??0?00?1?????01000??????????00100000?1111101?1?0000000000?00000001000000???????11010100101000?00??0???????????????????????????02--??0?0100001000?0???1010??????1?1100011?110010111?01010??000010000010001001?10??????????0110011000100?10?00001?0011???????????????????0???????????

Eocursor_parvus ??????????????????????????????????????????????????????????00001?????????????????????????????0?2211001000?0?????????????????0??0??????????1????0??1?????100?001??1001?0?2011?11??1???????0?0001????0??010?0?01?1?0??????01101??????1??1?0?1001??????????????????????????02--1101020000?110????01-?0100?002?110002101200001111110100?000020000?20101001?101??????????1?????????????????????????11?????00??????????0----------?

Silesaurus_opolensis ??00020000000010?0000?0000000001??000?0??00?00010?0000?1000?00100000?1100020000?0001???10???0102110010000000?0101100010001100?100000100121?0?000110--00112100100000101010010101111101000000000000010000000?000110000???01101010010100000010010-00??0?00??0?????????????02--1001010000100010--1101010000011012011101110[0 1]001100101?0110001001011000100111011?10????-0110011010100-10-01----20111110??00010101001210----------?

Pisanosaurus_mertii ??????????????1?00??0?1??0???????????????????????????????????????????????????????????????????????????????????????????????????????????0???1??????????????000001?012??00?2111111??1????0?0???0000??????0???????????????????????????????????????????????????????????????????????0????????000???????????????2????????????????????1010??0000?00000100010011101????????-0110??111?1?0-10-01----211111?????0?1?10??????0----------?

Heterodontosaurus_tucki 010000001010011000000110000000010000000010110001000000000020001000000130001000000001?011???1012211001110000000100110??000??00?0???0??001210010011111001?0[0 2]00010012010[0 2]12100111111100000001000000000?2011?0?011100001?1-01101?0-0101?01001100100010001100010000111201000-2--1100021000111010--01--0100000211?0?????-20?0?1011110100?0000200001101??00110?1111000?0-?110??1?1?1??-11-01----??111110000?0101010-1200----------2

Lesothosaurus_diagnosticus 0100030000000110000000100000000100000000?0110001000000000[0 1]00001000000130000000100001001?0001012211001000000000?00?10???00??00100?00??00121001001111100110000010012010002011011111??000000100000???0?001??0??111?0??1???011010?????1??100?10000?0100010???000?0?0??0100002--1101021000111010--01--01000002111000210120000101111?1000000020000020101??111??1??????0-01100-11101-0-10-01----211111100000010101???-00----------?

Scutellosaurus_lawleri ??????????????1??0??0?1??00???0??????????00????10???0????12???1??????????0000???0?0?????0????????????????0????????1??????????????????0?12?????????????110[0 2]0???????????02011011??10??00?00??0?0000?0??000???0???00?00?1-?1?0??0-00?00000011001000?????????0?????????????02--110102?1001110?????1--00??????111000?1?120000?0111101?0000002000002010-001?1011???????-01100111101-0-1?-01----211?10????????????????01-????000???

Herrerasaurus_ischigualastensis 01000100100100000000?0000000000?0000010000?10001000000000000001000000130001000000001??1??0?10?22?1011000?0000000??1????00??0?100?????00121001001110--00??00000?00001?001000001111100?01001100100001?2000001000001011?1-?1?01??????1??110110010000000112011001110130110102--1000021000100011101101010200021010102102100101011110101?0000200100101010011101111011?0-01101111101-0-10-01----201111100000011101?01100----------?

Staurikosaurus_pricei ?????????????????????????????????????????????????????????????????????????????????????????????????????????????????????????????????????????1????????????00000?00?000?1?001000001??1???????01?00??0????000000100?00101????????????????????????????????????????????????????02--1000021000100?1110110101?1?00?11?000210110010111111010??0000200100100010011101????????????????????????????-??????????????????????????0----------?

Eoraptor_lunensis 010001001012000000000?000100000??001111000?100010?00000?0?0??01000001130001000000001?01?????0??21?01?000???0?0??????????0????????????0012100?001110--000?00000????0??001?00?01011???0??001?0??0000?020000010?000?0?1?1-01001?0-?10100110110010001000111??10011110??110102--100112?001100011--?1000101000?11??0??1?110?00????110?0???000[1 2]0010?10001001?1011110110?-?1?0???1?01-0-10-0?-???2?111110000001?1010?1?00----------1

Saturnalia_tupiniquim ?????????????????0??0????????????????11????????????????1??????????????????????????????????????????????????0????????????????0??????????1??????????????????0???????????002001001??1??????0??100000?01?000000?000001001???0100100-01010010001101??01??0???????????????????02--10010110001000110011000102100210100021021001011111101?1?000020000111101001110111101100-01101111101-0-10-01----201111100000011101001?00----------?

Plateosaurus_engelhardti 10101200100100100000000010100001000111100001000100000001000000100000013001000000000100110001012211001000000000000110010001100?00?020?01121001?01110--00101000110000110020110011111100010011000000010000000100100100001-0100100-010100100111010001000101010111111120100?02--10000210001000110011000102100210100021021000011111101010000020000011101001110111101100-01101111101-0-10-01----201110100000011101001100----------?

Efraasia_minor ?01011001001001000000000?0000001?00??110?001???10?0000?10???0??????00????1?00000000?????????012211001000000000000110???00?100100?0???0112100?001?????0000000?1?000?1?002001001??11??10?001100-0-00100000001000001001?1-01001?0-01010010011101000100010?01011?1?11?0100?02--1000021000100010--110001021002101000210210000111111010100000200000111010011101111011?0-0110??111?1-0-10-01----201110100000011101001100----------1

Tawa_hallae 10000000101?00000000010?00000001000??1100001000100000000000???100001?130000000?00001??110??101221101?000000000000-1?1?0001100100000010012100?001110--000000000?000?1?001000001??1110111111110100001000000010?000?011?1-01101??????1??11011001000000011200?001110120110102--1001120001100??110?1010??1???2?1200022011000012110101011000020011010001001110111101100-01101?11101-0-10-01----211111100000010101?01200----------1

Coelophysis_bauri 10101100101?0000?0000?01010000010?010110000100010?000001000?001000011130001000??0001001100010122110110100000?0100110??000?10??00?000?00121?01001110--000?00000?00001?0010000011111101111111110000010000100?01110101111-01101?0-010100110110010001000112111001111130?10112--11021211111000111011?10101110211200021021001012110101?11000021011111101001110111100100-01100111101-0-11-01----211111110001010??10012?0------0??-?

Dilophosaurus_wetherelli 10101100?01?0000?00001??000100010?0??110???100010?0000000??????0?0011?300000000?000?0????00?0122110110000000?11001100?0?01100?00102??00121?01101??0--00000001000?00110010000011111000111111200000010000000?011101??1?1-11101?100101001100100100010?0112??10011?1120?10112--11021210111000?110?1010101111210200021?12000012110101?110?0021011121101001110111100100-01100111101-0-10-01----2111111100010101?1001200----------1

Allosaurus_fragilis 101012001001010-000001001021000100111110000100010000000000000010000011300000000000010111000101221101100000000110011????00??00000??2??00121001101110--00000001000000110010000011111001010111200000010000000101111111111-001010100101001101100100010001121111011111-1---102--1102021011100-1101110201011112102000220020000101101010110000210111211010011101111001?0-02100-11101-0-10-01----211110110000010101001200----------1

Velociraptor_mongoliensis 101011001000010100000?0000010001-001111-00010001000000010000?01000000131000000000001011100010122110?10000001011?01111???0??0[0 1]?00?????00121001001110--000000000?000011001000001111???????????????0010?0010011111110-111-00101?110101001001100100000000121110000111-1---102--11020210111100111011?200001002102010220-200001011010101100002101-12110-001?00?111????--0210??111?1-0-11-01----?11110110000010101001200----------1

CPEZ-239b ?????1?0000?00???0?0?00?0010?11??00?0???00?0?00?0?1?000?0?0?0?1???????????????000????????????????????????????????????????????0??????????????????????????????????????????????????????????????????????????????????????????????????????????????????????????????????????????????????????????????????????????????????????????????????????????????????????????????????????????????????????????????????????????????????????????????
