# Supplementary figures and images for: New insights on Prestosuchus chiniquensis Huene, 1942 (Pseudosuchia, Loricata) based on new specimens from the “Tree Sanga” Outcrop, Chiniquá Region, Rio Grande do Sul, Brazil
Source: PeerJ. 2016 Feb 1;4:e1622. doi: 10.7717/peerj.1622 (PMC4741083; doi:10.7717/peerj.1622)

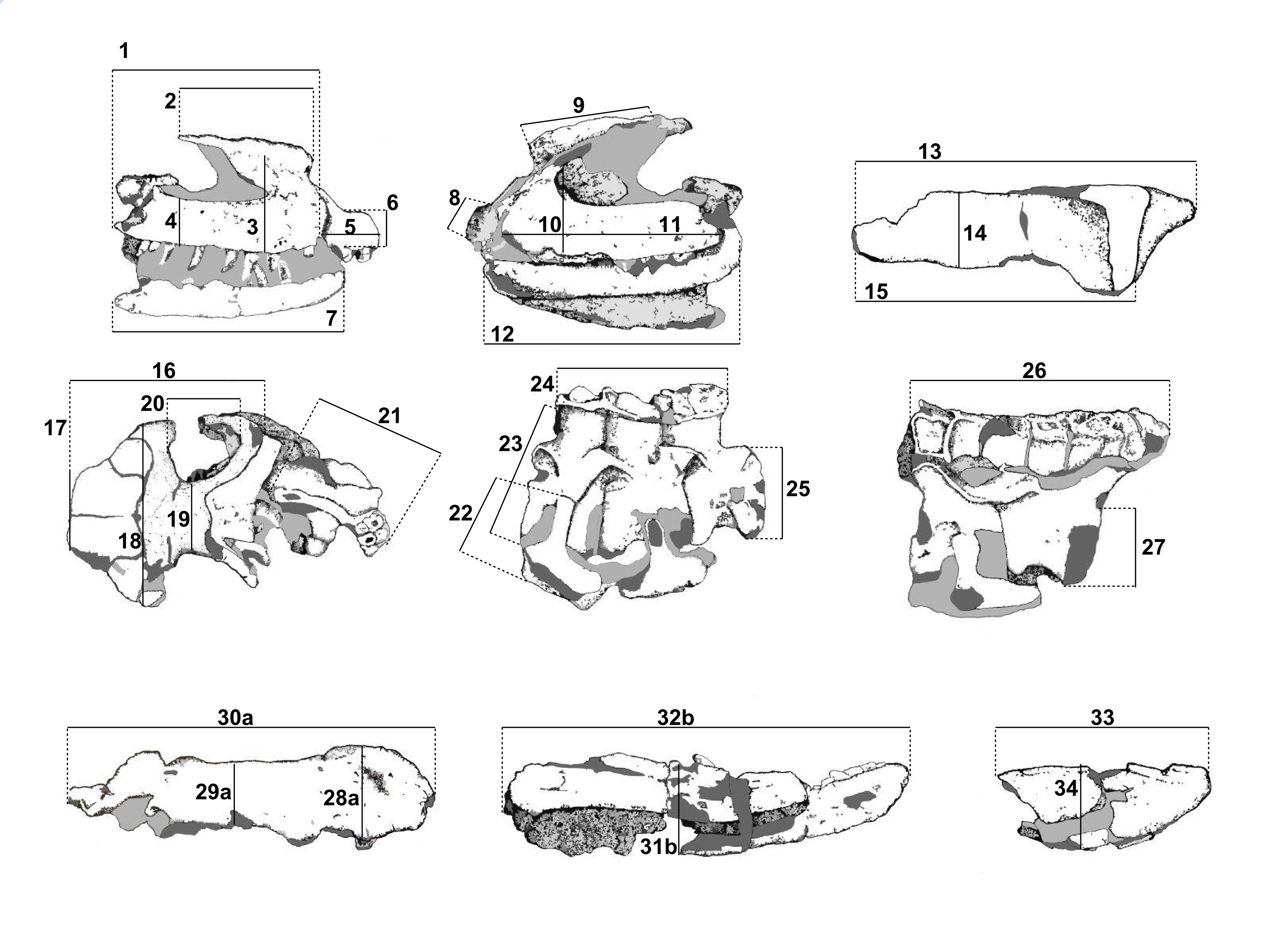

Supplement: Supplemental Information 2 — Measurement parameters 1. [file peerj-04-1622-s002.png]

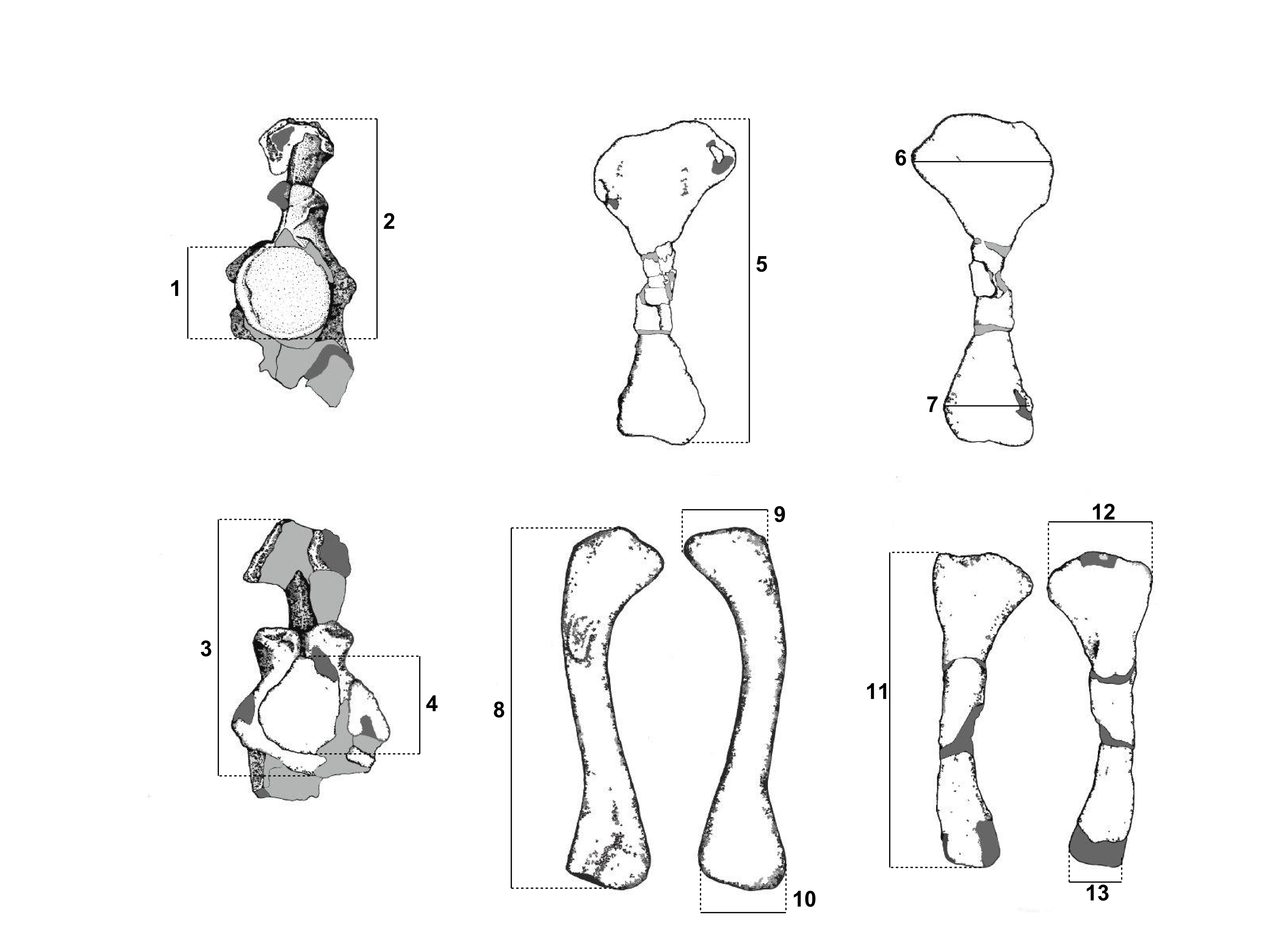

Supplement: Supplemental Information 3 — Measurement parameters 2. [file peerj-04-1622-s003.png]
