# Supplementary material for: New insights on Prestosuchus chiniquensis Huene, 1942 (Pseudosuchia, Loricata) based on new specimens from the “Tree Sanga” Outcrop, Chiniquá Region, Rio Grande do Sul, Brazil
Source: PeerJ. 2016 Feb 1;4:e1622. doi: 10.7717/peerj.1622 (PMC4741083; doi:10.7717/peerj.1622)
Supplement: Supplemental Information 4 — Measurement Table. [file peerj-04-1622-s004.docx]

| **Number** | **Measurement** | **Value**  **(in mm)** |
| --- | --- | --- |
| 1 | Length of the right maxilla | 186 |
| 2 | Length of the right nasal | 125 |
| 3 | Height of the ascending process of the maxilla close to the anterior border of the antero-orbital fenestra | 75 |
| 4 | Height of the right maxilla | 38 |
| 5 | Length of the right premaxilla | 44 |
| 6 | Height of the right premaxilla | 26 |
| 7 | Length of the right mandibular ramus | 204 |
| 8 | Height of the left premaxilla | 26 |
| 9 | Length of the left nasal | 92 |
| 10 | Height of the ascending process of the left maxilla | 71 |
| 11 | Length of the left maxilla | 167 |
| 12 | Length of the left mandibular ramus | 201 |
| 13 | Length of the left lacrimal and prefrontal | 123 |
| 14 | Heigth of the left lacrimal | 23 |
| 15 | Length of the left lacrimal | 95 |
| 16 | Length from the preserved borders of the frontals to the posterior border of the right parietal | 110 |
| 17 | Width of the anterior border of the Series B | 45 |
| 18 | Width between the right and left postorbital | 101 |
| 19 | Width of the central portion of the parietal | 26 |
| 20 | Length of the right supratemporal fenestra | 36 |
| 21 | Length of the axis neural apophysis | 79 |
| 22 | Height of the right cervical rib of vertebrae C8 | 63 |
| 23 | Height of the vertebrae C8 up to the base of the osteoderm | 99 |
| 24 | Length of the osteroderm row | 113 |
| 25 | Height of the centrum of vertebrae C6 | 47 |
| 26 | Length of the osteoderm row | 152 |
| 27 | Height of the centrum of vertebrae C4 | 40 |
| 28a | Anterior height of the isolated right maxilla | 40 |
| 28b* | Anterior height of the isolated left maxilla | 50 |
| 29a | Posterior height of the isolated right maxilla | 27 |
| 29b* | Posterior height of the isolated left maxilla | 32 |
| 30a | Length of the isolated right maxilla | 173 |
| 31b* | Length of the isolated left maxilla | 140 |
| 31a* | Height of the isolated right mandibular ramus | 41 |
| 31b | Height of the isolated left mandibular ramus | 33 |
| 32a* | Length of the isolated right mandibular ramus | 193 |
| 32b | Length of the isolated left mandibular ramus | 194 |
| 33 | Length of the fragment of a posterior portion of a left mandibular ramus | 122 |
| 34 | Height of the fragment of a posterior portion of a left mandibular ramus | 37 |

| **Number** | **Measurement** | **Value**  **(in mm)** |
| --- | --- | --- |
| 1 | Height of the centrum of vertebrae C8 | 40 |
| 2 | Total height of vertebrae C7 | 116 |
| 3 | Total height of vertebrae C6 | 185 |
| 4 | Height of the centrum of vertebrae C6 | 38 |
| 5 | Length of the humerus | 195 |
| 6 | Width of the proximal epyphysis of the humerus | 84 |
| 7 | Width of the distal epyphysis of the humerus | 59 |
| 8 | Length of the femur | 325 |
| 9 | Width of the proximal surface of the femur | 81 |
| 10 | Width of the distal surface of the femur | 71 |
| 11 | Length of the tibia | 223 |
| 12 | Proximal width of the tibia | 72 |
| 13 | Distal length of the tibia | 39 |
